# Novel Essential Role of Ethanol Oxidation Genes at Low Temperature Revealed by Transcriptome Analysis in the Antarctic Bacterium *Pseudomonas extremaustralis*

**DOI:** 10.1371/journal.pone.0145353

**Published:** 2015-12-15

**Authors:** Paula M. Tribelli, Esmeralda C. Solar Venero, Martiniano M. Ricardi, Maria Gómez-Lozano, Laura J. Raiger Iustman, Søren Molin, Nancy I. López

**Affiliations:** 1 Departamento de Química Biológica, Facultad de Ciencias Exactas y Naturales, Universidad de Buenos Aires, Intendente Guiraldes 2160, C1428EGA Buenos Aires, Argentina; 2 IQUIBICEN, CONICET, Buenos Aires, Argentina; 3 Instituto de Fisiología, Biología Molecular y Neurociencias (IFIBYNE-CONICET), Facultad de Ciencias Exactas y Naturales, Universidad de Buenos Aires, Buenos Aires C1428EGA, Argentina; 4 Novo Nordisk Foundation Center for Biosustainability, Technical University of Denmark, Hørsholm, Denmark; National Renewable Energy Lab, UNITED STATES

## Abstract

Temperature is one of the most important factors for bacterial growth and development. Cold environments are widely distributed on earth, and psychrotolerant and psychrophilic microorganisms have developed different adaptation strategies to cope with the stress derived from low temperatures. *Pseudomonas extremaustralis* is an Antarctic bacterium able to grow under low temperatures and to produce high amounts of polyhydroxyalkanoates (PHAs). In this work, we analyzed the genome-wide transcriptome by RNA deep-sequencing technology of early exponential cultures of *P*. *extremaustralis* growing in LB (Luria Broth) supplemented with sodium octanoate to favor PHA accumulation at 8°C and 30°C. We found that genes involved in primary metabolism, including tricarboxylic acid cycle (TCA) related genes, as well as cytochromes and amino acid metabolism coding genes, were repressed at low temperature. Among up-regulated genes, those coding for transcriptional regulatory and signal transduction proteins were over-represented at cold conditions. Remarkably, we found that genes involved in ethanol oxidation, *exaA*, *exaB* and *exaC*, encoding a pyrroloquinoline quinone (PQQ)-dependent ethanol dehydrogenase, the cytochrome c550 and an aldehyde dehydrogenase respectively, were up-regulated. Along with RNA-seq experiments, analysis of mutant strains for *pqqB* (PQQ biosynthesis protein B) and *exaA* were carried out. We found that the *exaA* and *pqqB* genes are essential for growth under low temperature in LB supplemented with sodium octanoate. Additionally, p-rosaniline assay measurements showed the presence of alcohol dehydrogenase activity at both 8°C and 30°C, while the activity was abolished in a *pqqB* mutant strain. These results together with the detection of ethanol by gas chromatography in *P*. *extremaustralis* cultures grown at 8°C support the conclusion that this pathway is important under cold conditions. The obtained results have led to the identification of novel components involved in cold adaptation mechanisms in this bacterium, suggesting for the first time a role of the ethanol oxidation pathway for bacterial growth at low temperatures.

## Introduction

Bacterial adaptability to an environment is the result of complex mechanisms that entail the response of individual genes or operons and intricate regulatory networks that coordinate the control of entire metabolic pathways [1]. Survival in extreme environments requires additional features at nearly all levels of cell function. In the case of cold environments, the low temperatures and the presence of ice exert severe constraints on living organisms, including decreased water availability and molecular diffusion rates, reduced biochemical reaction rates, stabilization of inhibitory nucleic acid structures, presence of ice crystals, increased solubility of gases, production of reactive oxygen species (ROS) and reduced fluidity of cellular membranes [2,3]. Microorganisms that are able to survive and grow in cold and freezing environments should thus present physiological adaptations to cope with these conditions, including expression of cold shock proteins, membrane modifications and ribosome rescue [3,4]. *Pseudomonas* species are metabolically versatile and energy can be obtained from different central and secondary pathways such as Entner-Doudoroff route, periplasmic glucose oxidation (involving *gcd* and *gad* genes), ethanol oxidation (including *exaA*, *exaB* and *exaC* genes), pyruvate and arginine fermentation (including *ack*, *pta*, *adhA* and *ldhA* and *arcDABC* genes respectively)[5–7]. However, metabolic features associated with growth in cold conditions and the relevance of the different pathways has not yet been totally elucidated.

*Pseudomonas extremaustralis* is a bacterium isolated from Antarctica [8] that shows high stress resistance connected to the production of high amounts of polyhydroxyalkanoates (PHA), mainly as polyhydroxybutyrate (PHB), a short chain length PHA but also is able to produce medium chain length PHA [9]. Genome analysis has revealed the presence of several fitness-related traits [10]. In this bacterium, PHB accumulation is essential for cold growth, freezing survival and important in oxidative stress resistance [11]. Additionally, PHB contributes to the development of a planktonic life style at low temperatures [12]. In comparison with other *Pseudomonas* species such as *P*. *putida* KT440, *P*. *aeruginosa* PAO1 and *P*. *protegens* Pf5, *P*. *extremaustralis* grows faster and reaches higher biomass yields at low temperatures [10–12]. Additionally, previous work has shown that micro-aerobic metabolism is relevant for this bacterium, and that the anaerobic global regulator, Anr, is involved in novel functions such as PHB metabolism, redox balance, oxidative stress resistance and biofilm development [13–15].

Genome-wide RNA-deep sequencing (RNA-seq) is a powerful tool to analyze gene expression and detect small regulatory RNA in bacterial species as well as to discover previously non-annotated genes [16,17]. This technology has been used to study transcriptome profiles under various conditions, for example in *P*. *aeruginosa* [18–20], *Listeria monocytogenes* [21] and *Escherichia coli* [22,23]. Although there are RNA-seq data in *P*. *putida* growing at 10°C [24], transcriptome analysis of a species isolated from a cold extreme environment has been performed only in *Planococcus halocryophilus* Or1, a bacterium that inhabits permafrost [25].

In this work, new insights for cold adaptation were explored by carrying out an RNA deep-sequencing analysis of *P*. *extremaustralis*, an Antarctic bacterium, to study genome-wide expression of genes under cold conditions along with mutant strain construction of genes related to ethanol oxidation pathway, *pqqB* and *exaA*.

## Materials and Methods

### Strains and culture conditions

*P*. *extremaustralis* [8] was used throughout the experiments. Cultures were grown in LB medium supplemented with 0.25% sodium octanoate (for PHA accumulation). Cultures were incubated under aerobic conditions (200 rpm) at 8°C or 30°C. OD_600nm_ was measured through time. To test ethanol oxidation, cultures were performed using E2 minimal medium [26] supplemented with ethanol (0.5% v/v).

### RNA extraction and RNA library preparation

Total RNA was isolated from 15 ml *P*. *extremaustralis* cultures using the Trizol method [18]. Cultures exponentially grown under 8°C and 30°C aerobic conditions were initiated at 0.05 and harvested at 0.5 OD_600nm_. RNA quality was analyzed on an Agilent 2100 Bioanalyzer (Agilent Technologies). As ribosomal RNAs (rRNAs) account for the vast majority of total RNA in a bacterial cell [27], rRNAs were removed by subtractive hybridization to enrich samples for mRNAs using the MICROBExpress Kit (Ambion) with the addition of 5S oligonucleotides, as previously described [18,28]. After each step, the samples were validated using an Agilent 2100 Bioanalyzer (Agilent Technologies), and the final concentration was measured using a Qubit 2.0 Fluorometer (Invitrogen). Directional libraries were prepared with the ScriptSeq v2RNA-Seq Library Preparation Kit (Epicentre) and sequenced using the Illumina HiSeq2000 platform with a paired-end protocol and read lengths of 100 nt. For each condition, duplicated independent RNA extraction and libraries were used.

### RNA-seq data analysis

The data were de-multiplexed by Beckman Coulter Genomics. Reads alignment in *P*. *extremaustralis* genome and transcript abundance levels were quantified using the reads per kilobase per million mapped reads (RPKM) with Rockhopper software [29], using default parameters. Concordance between replicates at 8°C and 30°C was verified by performing a Spearman correlation analysis of normalized counts (S1 Fig). Transcripts with less than 20 normalized reads were discarded from further analysis. Differential gene expression was considered as significant only when P<0.05 and Q<0.05 using Rockhopper software analysis. Functional enrichment of differentially expressed genes was determined using Blast2GO software by assigning the GO category to all genome sequences and to the differentially expressed genes [30].

### Quantitative Real Time PCR Experiments (RT qPCR)

Total RNA of *P*. *extremaustralis* was extracted from 6 ml of 0.5 OD_600nm_ aerobic cultures at 8°C or 30°C using the Total RNA Extraction Kit (RBC Biosciences). After treatment with DNaseI, cDNA was obtained using random hexamers (Promega) and AMV retrotranscriptase following the manufacturer’s instructions. At least three independent cultures were analyzed for each condition. RT qPCR was performed using a LightCycler (DNA Engine M.J. Research) and Real Time PCR mix (EvaGreen qPCR Mix Plus, no Rox). Different genes were analyzed using the following primers: *exaA* 5’ GAAGACTACATCGGCGTGCT 3’ and 5’ AATCCCAGACCTTCTCGCC’ 3; *erbR* 5’GGCGATCCAGCAGATTCTCA’ 3 and 5’ ATCTCCAGGGTGTAGGCGAT’ 3; *cspA* 5’TTGGCTTCATCACGCAGGA’3 and 5’ACAGGGACGGACGTTTTC’3; *azu* 5’GATCGACAAGAGCTGCAGGA’3 and 5’AGAAACCCGTAGTCCGTACCC ‘3 and *pelB* 5’ CGCCTGATGTGGCTCTATGT 3’ and 5’ATCTTCATAACCGGCGCTG ‘3. The 16S rRNA gene using primers 5’AGCTTGCTCCTTGATTCAGC’3 and 5’AAGGGCCATGATGACTTGAC’3 was used as reference for normalization of expression levels of target genes in each condition. The cycling conditions were as follows: denaturation at 95°C for 5 min, 40 cycles at 95°C for 25 s, 60°C for 15 s, and 72°C for 15 s, with fluorescence acquisition at 80°C in single mode. Relative changes in the expression of individual genes at 8°C and 30°C were obtained through the relative standard curve method [31].

### Generation of mutant strains

The *pqqB* mutant was identified during the construction of a transposon mutant library of *P*. *extremaustralis* using pUT mini-Tn5 and *E*. *coli* S17-1 as donor strain in a conjugation assay [32]. This mutant strain, unable to grow under cold conditions, was selected by plating transconjugants on LB agar supplemented with sodium octanoate (0.25%) and tetracycline (10 μg/ml) both at 8°C and 30°C. To identify interrupted genes, a two-step PCR strategy was performed as described before [33]. The final PCR product was purified and sequenced (Macrogen, Korea).For the construction of the ethanol dehydrogenase PQQ-dependent mutant strain (*exaA* strain), a PCR crossover deletion method was used, as described previously [34]. PCR mixtures were carried out according to [35]. The thermal cycle profile was 45 s at 94°C, 30 s at 60°C, and 30 s at 72°C (for a total of 34 cycles) and a final extension step for 10 min at 72°C. For the first PCR reaction, for the amino-terminal sequence, primers Hydro2up and HydroRlow (5-ATATGGCCGAGATGATCCGC-3 and 5-ATGACCCGATAGTAAAGAGCCCGGATCCCTGATTCTTGGGC-3) were used. For the carboxy-terminal sequence, the degenerate primers AldhLow (5-GTTGGTGAAGTATTGACCGCTG-3) and AldhRup (5-GCCAAGAATCAGGATCCGGGCTCTTTACTATCGGGTCAT-3) were used. In the second step, the left and right fragments were annealed at their overlapping region and amplified by PCR as a single fragment, using the external primers to obtain a 450-bp final product. A BamHI site was generated in the middle of the fragment. The amplification fragment was cloned in pGEM-T Easy and subcloned in pBluescript KS- (Stratagene). The plasmid was cut with BamHI and ligated with a kanamycin (Km) cassette obtained from the plasmid pUC4 K (Pharmacia, San Francisco, CA, USA). The resulting plasmid pBAlcD, which does not replicate in *Pseudomonas*, was used to generate an almost complete deletion of the *exaA* gene (in which only 44 bp and 94 bp of the 5´ and 3´ end, respectively, remain) and an insertion of the Km cassette in the *P*. *extremaustralis* chromosome. Plasmid pBAlcD was introduced by transformation into competent cells of *P*. *extremaustralis* [36]. Transformants were selected by plating on LB agar containing 40 μg/ml of Km at 30°C. Clones unable to grow using ethanol at 30°C as the sole carbon source were selected.

For complementation experiments, *pqqBCDE* genes with 300 bp upstream from the ATG was cloned into pBBR1MSC-5 [32] and introduced into the corresponding mutant strain by electroporation [36].

### Estimation of ethanol metabolism

To assay ethanol dehydrogenase activity a mixture of p-rosaniline and NaHSO_3_, often referred to as the Schiff’s reagent, was used as described before [37]. Aldehyde indicator plates were set by promptly adding 4 ml of a freshly prepared p-rosaniline solution, 2.5 mg/ ml in 95% (v/v) ethanol and 50 mg of NaHSO_3_ to 200-ml batches of pre-cooled (45°C) LB agar, previously amended with 0.5% (v/v) ethanol or 0.25% (w/v) sodium octanoate. Most of the dye is immediately converted to the leuco form by reacting with NaHSO_3_ to produce a rose-colored medium that was dispensed into Petri dishes. The wild type strain, the *pqqB* and its complemented strain were cultured in LB supplemented with sodium octanoate and 10 μl drops of these cultures were incubated in p-rosaniline plates at 8°C or 30°C for 7 days and 1 day, respectively. Magenta-colored bacterial spots were considered positive for alcohol dehydrogenase activity while white spots were considered negative [37]. As was described, the leuco dye acts as a sink, reacting with acetaldehyde to form the Schiff base which is intensely red, thus this reaction would be expected to shift the equilibrium for alcohol dehydrogenase toward aldehyde production that is soluble and diffused into the surrounding agar [37].

To estimate quantitatively alcohol dehydrogenase activity using p-rosaniline assay, absorption spectrum of leuco and shift base forms was determined. Two λ were selected: 548nm and 420nm in which a strong peak appears and a slight peak disappears respectively in magenta p-rosaniline form (S2 Fig). Three spots of each strain seeded in each condition, as described above, were recovered and suspended in 1 ml of physiological solution and homogenized with vigorously vortex during 10 min. One aliquot was used to determinate the colony forming units per ml (CFU/ml) in LB plates. Another aliquot (500 μl) was mixed with 500μl of absolute ethanol, in which p-rosaniline is soluble, and incubated for 90 min. After, the suspension was centrifuged for 5 min at 13,000 rpm. The supernatant was used to determinate the absorption spectrum (S2 Fig) and the absorbance at 548 nm and 420 nm was measured. The 548 nm/420 nm was calculated and normalized by CFU/ml. Finally we determined the p-rosaniline index (p-r index) as the result of OD 548nm/420nm/CFU/ml multiplied by the constant 10^9^. Five independent experiments were performed for each condition.

Ethanol in *P*.*extremaustrali’s* supernatant of cultures grown during 7 days at low temperatures in sodium octanoate LB was detected experimentally using a gas chromatography system (GC) (Agilent 7820, Santa Clara, CA). The GC system was equipped with a FID and an injector ALS7693A (Agilent). Separation of compounds was conducted on a 60 m HP-INNOWAX capillary column of 0.25 mm i.d using nitrogen as the carrier gas and a pre-column of inert silica (0.25μ). The injection volume was 1μl (1 mL of sample in the vial) and the flow rate was 1.5 ml/min. The injector temperature was 200°C with a split ratio of 40:1 and the FID temperature was 300°C. The oven temperature was programmed as follows: the column was held initially at 50°C for 5 min, then increased to 90°C at 10°C/min and held for 0.5 min. Absolute ethanol was used as standard. Chromatographic data were recorded and integrated using Agilent Chemstation software.

### Statistical analysis

The significance of the differences among strains in growth and p-rosaniline experiments was evaluated by the Student’s *t* test with confidence levels at >95% (*i*.*e*., *P*<0.05 was considered as significant).

## Results

### General features of the *P*. *extremaustralis* transcriptome profile at low temperatures

**Fig 1:**
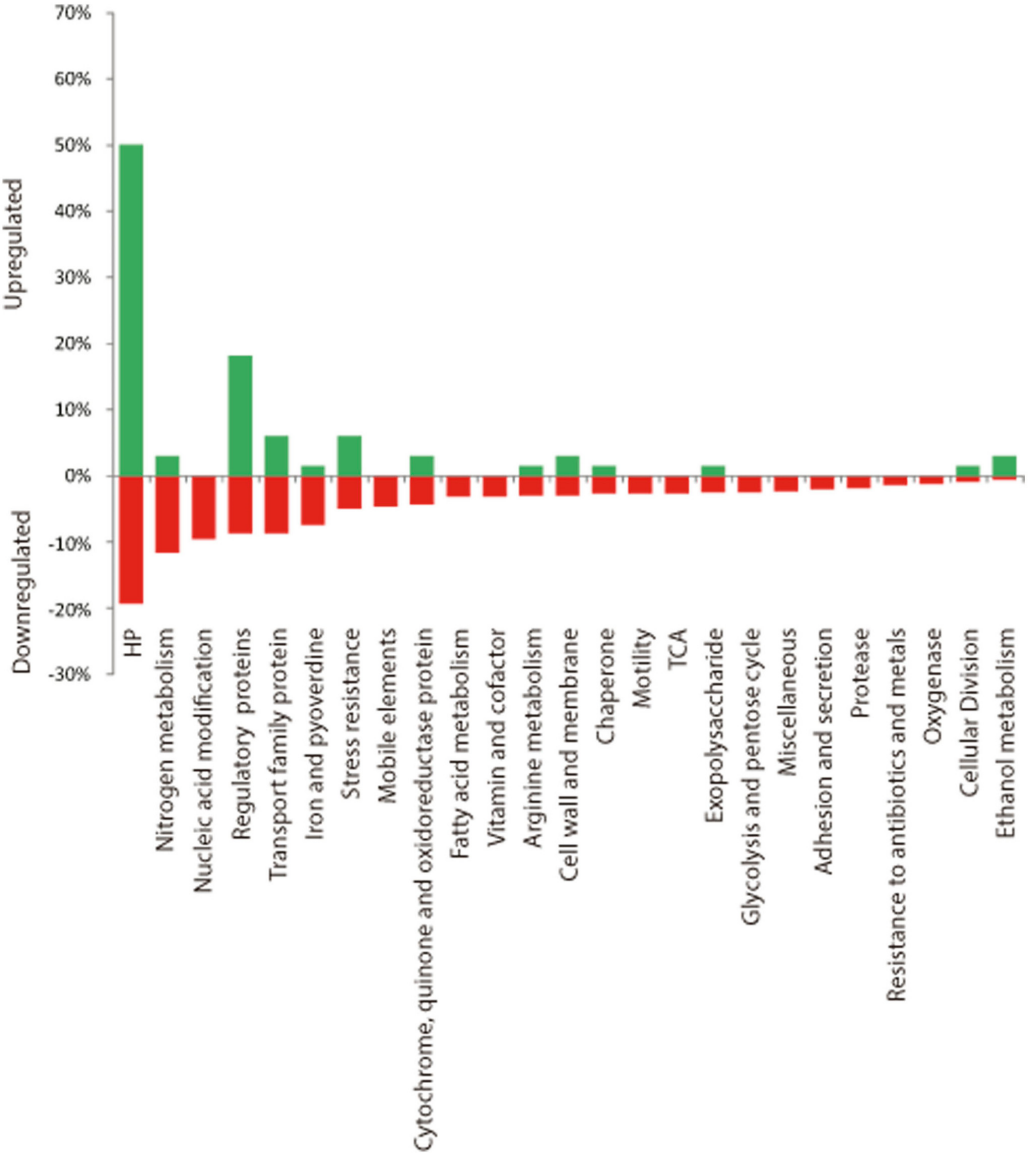
Classification of the significant differentially expressed genes under cold conditions into functional categories (Rockhopper P<0.05 and Q<0.05). Green and red bars represent up- and down-regulated genes, respectively.

The RNA expression profile of *P*. *extremaustralis* cultures growing at 8°C or 30°C at the early exponential phase (OD_600nm_ = 0.5) in LB supplemented with sodium octanoate revealed 5715 transcripts and 156 putative small regulatory RNAs. The Rockhopper software allowed us to identify genes differentially regulated at 8°C (relative to 30°C) with statistical relevance. In cold conditions, 623 genes were down-regulated and 67 were up-regulated relative to 30°C (P<0.05 and Q<0.05, S1 and S2 Tables). This technique also revealed the existence of 156 novel intergenic sRNAs. The differentially expressed genes were classified by function (Fig 1). Among the down-regulated genes were, cytochrome- coding genes such as *azu*, *cyoA*, *cyoB*, *cyoC*, *cyoD*, cytochrome c4 and B561, as well TCA associated genes (Fig 1, S1 Table). Cell division related genes (five genes) were repressed in the early exponential phase of growth at low temperatures in comparison to 30°C (Fig 1, S1 Table). Moreover, genes involved in pyoverdine biosynthesis (24 genes) and iron uptake, genes encoding other iron associated proteins (22 genes), and genes required for molybdopterin biosynthesis, (*mobA* and *mobB*) (S1 Table), were down regulated at low temperature. In addition, genes coding for Pel exopolysaccharide biosynthesis as well as motility genes including *flG*, *flgJH*, *flgK*, *flgL*,*fleQ*, *fliF*, *fliG*, *fliM*, *flhA* among others, were found to be repressed at low temperatures (Fig 1, S1 Table). Regarding nitrogen metabolism, the genes *potABCD*, *potFGHI*, *gabT* and *gabD*, involved in polyamine (putrescine and spermidine) transport and catabolism were also down-regulated (Fig 1, S1 Table). However, the gene coding for agmatinase, a key enzyme for putrescine biosynthesis from arginine, was up-regulated at cold conditions (Fig 1, S2 Table). Several genes encoding for chaperone function, including heat shock proteins, were repressed at 8°C, such as *groEL*, *groES*, *ibpA*, *dnaJ* and *dnaK* (Fig 1, S1 Table). Increased transcription in cold conditions was observed from a number of regulatory genes such as *rsmE*, *slyA*, *cpxR*, *algZ*, *rnk*, *cheY*, *cheC*, *erbR*, and from the major cold shock protein-coding gene, *cspA*, that could play a key role in low temperature adaptability (Fig 1, S2 Table). Interestingly, transcription of *exaA*, *exaB*, *exaC* and *erbR*, genes involved in the ethanol oxidation pathway, was also up-regulated in cold conditions (Fig 1, S2 Table).

Genes involved in osmotic resistance were found to be differentially expressed; sarcosine catabolism was repressed, while expression of an osmotically induced protein and of sodium glutamate symporter coding genes was up-regulated (Fig 1, S1 and S2 Tables).

**Fig 2:**
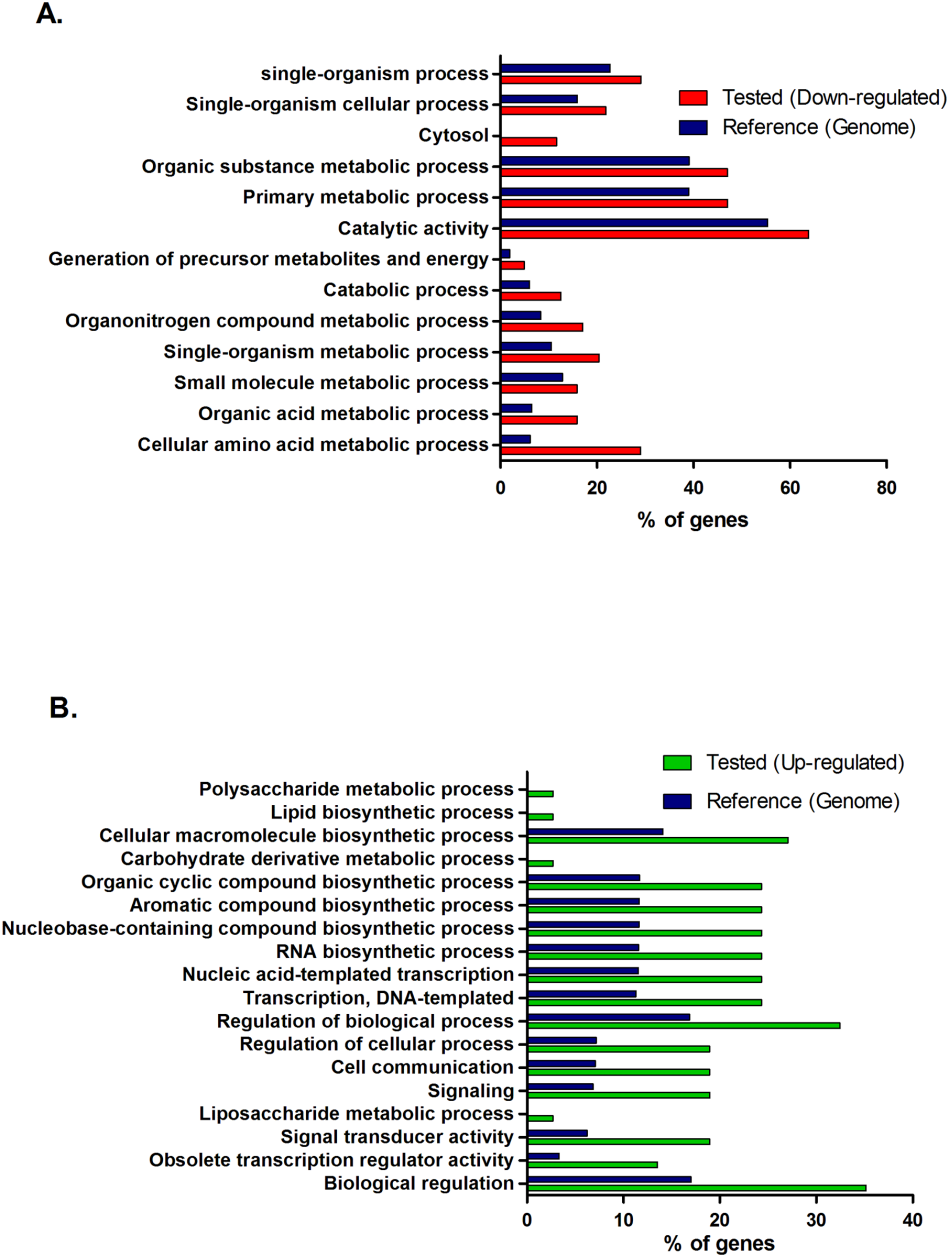
Functional enrichment of differentially expressed genes using Blast2GO software. A. Down-regulated genes. B. Up-regulated genes. P<0.05 using Fisher’s Test.

To analyze whether any functional category was over-represented in the group of differentially expressed genes (Rockhopper, P<0.05 and Q<0.05) a Blast2Go software analysis was carried out (S3 Table). This analysis showed that in the data set of up-regulated genes at cold conditions transcriptional regulation and signal transduction categories were over-represented along with aromatic and organic-cyclic biosynthesis compounds (Fisher’s test using Blast2GO, P<0.05, Fig 2). For down-regulated genes, primary metabolism as well as amino acid biosynthesis categories were over-represented (Fisher’s test using Blast2GO, P<0.05, Fig 2).

Some genes were chosen to validate RNA-seq results by using RT qPCR. Results showed that at cold conditions, *cspA* (2.5 fold), *erbR* (5.1 fold) and *exaA1* (7.35 fold) were up-regulated, while *pelB* (2 fold) and *azu* (7.5 fold) were down-regulated, in concordance with the RNA-seq analysis (S3 Fig).

Overall, the transcriptional profiles suggest that several genes up-regulated at low temperatures could be important for extended growth and for reaching higher biomass yields (OD_600nm_ = 7) as observed at 8°C after 72h of culture growth in *P*. *extremaustralis*.

### The ethanol oxidation pathway is up-regulated at low temperatures

**Fig 3:**
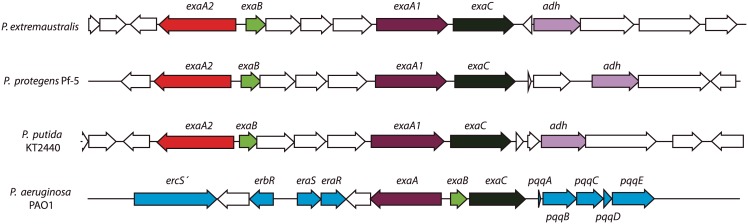
Organization of genes coding for ethanol oxidation in *P*. *extremaustralis* and other *Pseudomonas* species. Arrows indicate the direction of gene transcription and the relative size of each open reading frame (ORF).

The transcriptome analysis showed that the expression of all the genes involved in ethanol oxidation metabolism was up-regulated around 10 times at low temperatures (S2 Table). These genes included *exaA* encoding a PQQ-dependent ethanol dehydrogenase, *exaC* encoding an aldehyde dehydrogenase, and *exaB* coding for cytochrome c550 as well as *erbR* (or *agmR*), expressing a transcriptional regulator related to ethanol oxidation in *P*. *aeruginosa* (Fig 3, S2 Table) [6,38]. The genomic organization of these genes is similar to those found in *P*. *putida* KT2440 and *P*. *protegens* Pf-5, and different from that found in *P*. *aeruginosa* PAO1 (Fig 3). Two different alcohol dehydrogenase-coding genes, *exaA1* and *exaA2*, were identified, of which *exaA1* was located next to the *exaC* gene (Fig 3). In *P*. *extremaustralis* the aminoacid sequences of ExaA1and ExaA2 presented between them 51% of identity and 78% of similarity. The ErbR coding gene is located in the same genomic zone as the *exaA*, *exaB* and *exaC* genes, separated by 9 putative ORFs. Genes encoding proteins for PQQ biosynthesis, required for ExaA function, were located in another genome zone, forming a cluster containing *pqqFABCDE* with an organization similar to that observed in other *Pseudomonas* species (not shown).

RNA-seq results showed that only *exaA1* was up-regulated around 10 times at low temperatures (S2 Table), an observation further confirmed by qRT-PCR experiments using *exaA1* specific primers (S3 Fig). The extra copy of the *exaA* gene, *exaA2*, was oriented in opposite direction to *exaB* but showed low expression in the RNA-seq results at both 8°C and 30°C. Although it is not possible to rule out *exaA2* contribution, the results suggest that *exaA1* expression mainly contributes to the PQQ-dependent alcohol dehydrogenase expression at 8°C. Additionally, expression of the PQQ biosynthesis genes (*pqqA*, *pqqB*, *pqqC*, *pqqD*, *pqqF* and *pqqE*) at 8°C was not significantly different from that at 30°C. Similar expression at both temperatures was observed for the cytochrome c oxidase genes, which are involved in energy generation in ethanol oxidation in other bacteria [6].

### The ethanol oxidation pathway is essential for growth and survival at low temperatures

In addition to the transcriptome analysis, which showed an increase in the expression of genes involved in ethanol oxidation metabolism, a genetic approach was used to detect novel genes essential for cold adaptation. During the screening of a random mutant library, a clone unable to grow at low temperatures in agar plates was isolated, and the interrupted gene was identified as *pqqB*. The PQQ coenzyme is the cofactor of different enzymes such as PQQ-dependent glucose dehydrogenase, shikimate-quinate dehydrogenase and PQQ-dependent ethanol dehydrogenase encoded by the *exaA* gene. The *exaA*, *exaB*, *exaC* gene products and the PQQ coenzyme are involved in generation of energy when ethanol is used as the sole carbon source in *P*. *aeruginosa* PAO1 and *P*. *putida* KT2440 [6,38,39]. To assess the importance of the ethanol oxidation pathway at low temperatures, we further constructed an *exaA1* mutant strain. The presence of PHA granules was determined in both mutant strains due to the importance of this polymer on growth of *P*. *extremaustralis* at low temperatures [11]. Microscopic observations after Nile Blue staining of the *pqqB* and *exaA1* mutants cultured in LB supplemented with sodium octanoate showed the presence of PHA granules similar to those observed in the wild type strain (data not shown), ruling out an impairment in PHA biosynthesis which is essential for cold growth in *P*. *extremaustralis*. To confirm the ability of *P*. *extremaustralis* to grow using ethanol as carbon source in minimal medium and the essential role of the *pqqB* and *exaA1* genes in this metabolism, cultures of the wild type and both mutant strains were tested at 8°C and 30°C. The wild type strain was able to grow using ethanol as the sole carbon source, reaching OD_600nm_ 4.6 ± 0.6 at 30°C after 30h and OD_600nm_ 1.3 ± 0.3 at 8°C after 72h of culture, while the *pqqB* and *exaA1* mutant strains showed severe growth defects. The *pqqB* mutant was able to grow only at 30°C after 30h (OD_600nm_ values of 0.6 ± 0.1), showing significant differences (P<0.05) when compared to the wild type strain, whereas the *exaA1* mutant showed a slight growth only after 50h (OD_600nm_ 0.3±0.1). These results confirmed that *P*. *extremaustralis* was able to use ethanol as the sole carbon source at both 8°C and 30°C and the importance of *exaA1* and *pqqB* in this metabolic pathway.

**Fig 4:**
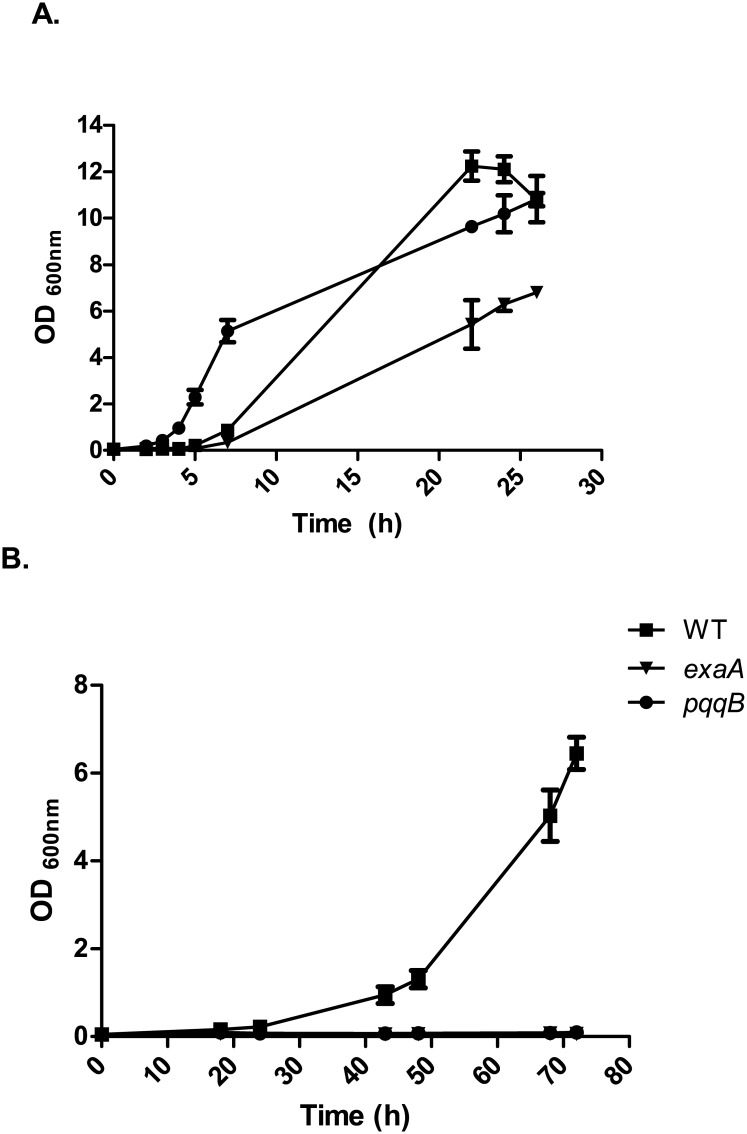
Growth of the wild type (WT), *ppqB* and *exaA* strains. A. Growth at 30°C. B. Growth at 8°C. Values represent the mean ± standard deviations (SD) from three independent cultures.

Transcriptome data showed that the *erbR*, *exaA1*, *exaB* and *exaC* genes were up-regulated at 8°C in the exponential phase. To test the physiological importance of the ethanol oxidation pathway in cold conditions, we carried out growth experiments at 8°C and 30°C. At 30°C, the wild type and the mutant strains were able to grow using LB supplemented with sodium octanoate and presented non-significant OD_600nm_ differences after 26h of culture (Fig 4, P>0.05). The growth rate at 30°C was 0.8±0.1 for the wild type strain and 1.1±0.2 and 1.1±0.5 for the *pqqB* and *exaA*, respectively. At cold conditions, the wild type reached an OD_600nm_ of 6.45 ± 0.63 of culture (μ = 0.33±0.05), while the *pqqB* and *exaA1* mutant strains were unable to grow after 72 h (Fig 4). Complementation with a wild-type allele of *pqqB* carried on a plasmid (pBBR1MSC-5) introduced into the mutant strain resulted in an OD_600_ of 1.96±0.22 with a μ value of 0.05±0.01. A control experiment with the wild type strain carrying the vector pBBR1MSC-5 without the *pqqB* gene showed a slight decrease in growth in the presence of gentamicin at low temperatures (OD_600nm_ of 5.68±1.19 after 72h culture). The growth of the complemented strain at cold conditions differed significantly from that of both the wild type and *pqqB* mutant strain (P<0.05), thus indicating that the complementation only partially restored the wild type phenotype. The results showed that *exaA1* and *pqqB*, both involved in the ethanol oxidation pathway, are essential for bacterial growth at cold conditions.

### *P*. *extremaustralis* is able to produce and oxidize ethanol

**Fig 5:**
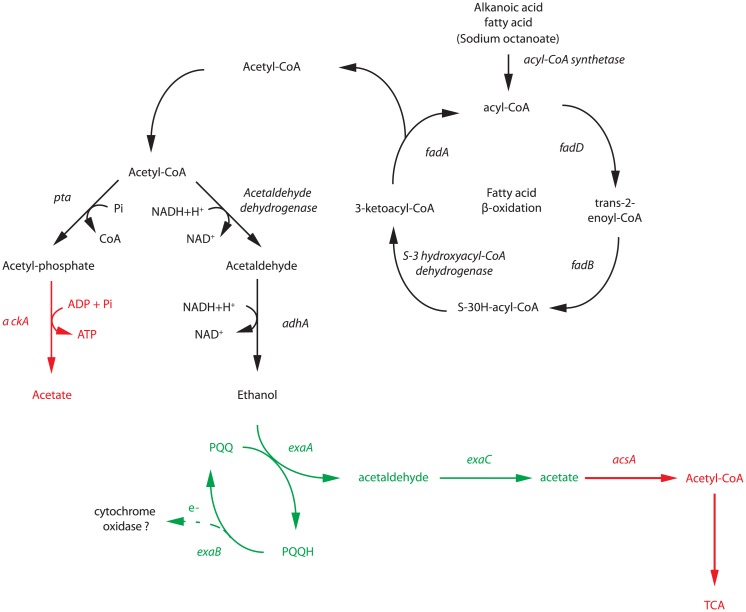
Relationship between ethanol metabolism and the ß-oxidation pathway. Solid black lines and names represent genes without differences in their expression. Solid red lines and names represent down-regulated genes and green solid lines and names indicate up-regulated functions under cold conditions. Dashed lines show probable relationships.

**Fig 6:**
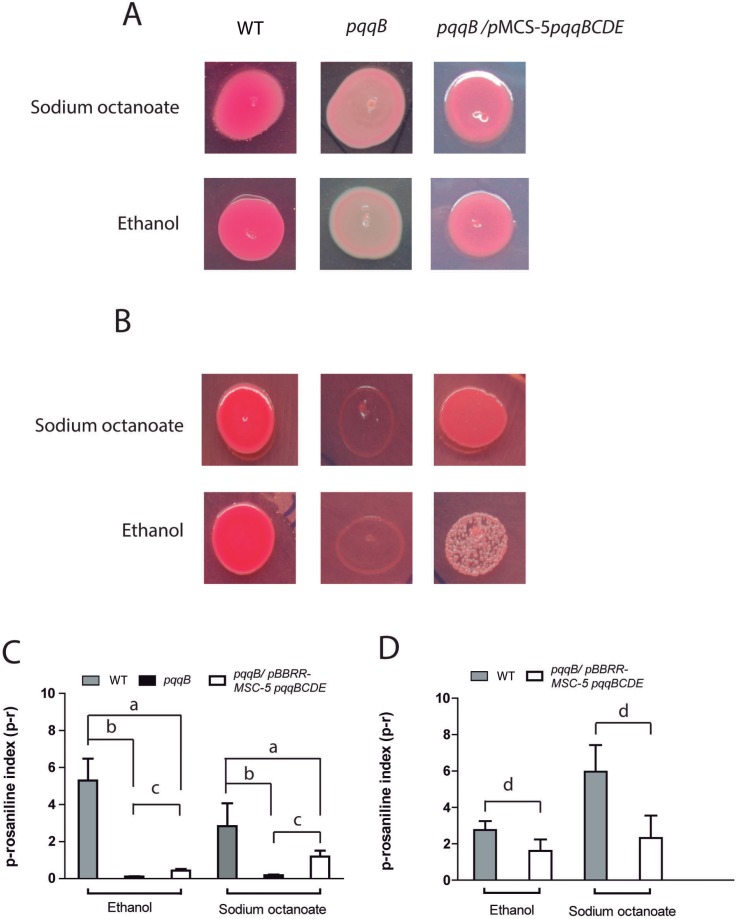
Estimation of alcohol dehydrogenase activity using p-rosaniline plate assay in wild type (WT), *ppqB* and *pqqB*/pBBR1MCS5 *pqqBCDE* strains. Activity was considered as positive in magenta colonies, while white colonies were considered as negative. A. Plates incubated at 30°C during 24 h. B. Plates were incubated at 8°C during 7 days. C. Determination of p-rosaniline index at 30°C. D. Determination of p-rosaniline index at 8°C. The asterisk (*) denotes significant differences (*P*<0.05) between strains (indicating by connector lines) using the Student's *t* test.

The up-regulation of genes involved in ethanol oxidation as well as the essential role of *pqqB* and *exaA* in the growth and survival at cold conditions raises the question whether ethanol is produced for further oxidation when sodium octanoate is used as carbon source. As a fatty acid, sodium octanoate is metabolized through the β-oxidation pathway, which includes several steps (Fig 5). The transcriptome analysis showed that although the expression of genes encoding enzymes involved in this pathway at 8°C was not different from that at 30°C, all enzymes were actively expressed at both temperatures. β-oxidation produces acetyl-CoA, a molecule that can be metabolized to acetaldehyde by the enzyme acetaldehyde dehydrogenase (acylating) in a reversible way. In the *P*. *extremaustralis* genome, we found three copies of genes encoding acetaldehyde dehydrogenase, two of which were expressed similarly at both temperatures, and the third of which showed no expression. The last step towards ethanol production is the reduction of acetaldehyde to ethanol, catalyzed by ethanol dehydrogenase as a branch of pyruvate fermentation pathway which is functional in *P*.*extremaustralis* [10]. We found eleven genes encoding proteins with high homology to ethanol dehydrogenase. Among them, four were not transcribed in the tested conditions, one was down-regulated at cold conditions, and the remaining six were expressed equally at 8 and 30°C. To test experimentally the possibility of ethanol production in LB supplemented with sodium octanoate we carried out a p-rosaniline assay in which ethanol dehydrogenase activity is detectable. It was observed that wild type *P*. *extremaustralis* displayed alcohol dehydrogenase activity at both 8°C and 30°C, showing magenta bacterial spots in LB agar plates supplemented with sodium octanoate (Fig 6A and 6B) and values of p-rosaniline index (p-r) of 5.97 ± 1.46 and 2.82 ± 1.33, respectively (Fig 6C and 6D). The *pqqB* strain was growing only at 30°C with white color bacterial spots and showed a significant decrease (near 25 fold, P<0.05) in the p-r index in comparison with the wild type strain (Fig 6A–6C). In the complemented strain, growth at 8°C was restored, showing light magenta bacterial spots (Fig 6A and 6B). The p-r index in LB plus sodium octanoate culture medium was 2.32 ± 1.23 and 1.18 ± 0.80 at 8°C and 30°C respectively, indicating that the complementation only partially restored the wild type phenotype since significant differences between the complemented strain and both wild type and mutant strains were observed (Fig 6C and 6D, P<0.05). Control experiments for all strains using LB agar plates supplemented with ethanol were also performed at both temperatures (Fig 6A–6D). These results suggest that in the wild type the acetaldehyde production is mainly dependent of a PQQ dependent alcohol dehydrogenase.

Additionally, ethanol was detected after 7 days of growth at 8°C in sodium octanoate supplemented LB cultures of *P*. *extremaustralis* by gas chromatography (S4 Fig).

Although we cannot rule out posttranscriptional regulation, genomic and transcriptomic analysis along with p-rosaniline assays and ethanol detection showed that *P*. *extremaustralis* has the functional genetic information for ethanol production as well as for its subsequent utilization through genes encoding proteins involved in the ethanol oxidation pathway (Fig 5).

## Discussion

In cold conditions, several changes in cellular physiology occur in bacteria, such as a decrease in membrane fluidity and stabilization of the secondary structures of nucleic acids, leading to a reduced efficiency of RNA transcription, translation and degradation [40]. Efficient cellular responses to these and other constraints contribute to survival and growth under cold conditions. *P*. *extremaustralis* is an Antarctic psychrotolerant bacterium able to survive cold and freezing [11,12]. In comparison with other well studied *Pseudomonas* species such as *P*. *putida* KT2440, *P*. *protegens* Pf-5 and *P*. *aeruginosa* PAO1, *P*. *extremaustralis* grows faster and reaches higher biomass at low temperature [10]. For these reasons, it is a good model to find novel mechanisms to better understand bacterial survival under cold conditions. The RNA expression profile of *P*. *extremaustralis* at early exponential phase showed that in cold conditions, 623 genes were down-regulated and 67 were up-regulated relative to 30°C. Additionally, we found 156 novel intergenic sRNAs in the genome of *P*. *extremaustralis*, including some conserved sRNAs, such as 6S RNA, 4.5S rRNA, RNase P RNA, tmRNA, PrrF1/2, RsmZ, RsmY and CrcZ [41].

The analysis of the RNA-seq profile showed that some pathways had expression patterns similar to those previously described using other genome-wide approaches, e.g. the proteomic study of the Antarctic bacterium *Pseudoalteromonas haloplanktis* [42] and the transcriptomic analysis of the reference strain *P*. *putida* KT2440 [24], thus indicating that some cellular function could be widespread among bacteria able to grow in cold conditions despite the origin of their isolation.

Oxidative stress resistance is highly important for bacterial adaptability and several mechanisms to cope with oxidative stress have been described including a metabolic reprograming towards NADPH production [43]. At low temperatures oxidative stress becomes more relevant since it has been demonstrated that ROS production is increased in these conditions [3]. In *P*. *extremaustralis*, our data show that genes encoding proteins related to oxidative stress defenses such as alkyl hydroperoxide reductase, glutathione peroxidase, OxyR and superoxide dismutase are repressed under cold conditions. Although these observations seem contradictory, similar results have been described for proteins of the oxidative defense system in the cold-adapted *P*. *haloplanktis*, whereas in *P*. *putida* KT2240 no changes in oxidative stress response enzymes have been described [24,42]. Other antioxidative responses include putrescine and spermidine accumulation [3,44] and alginate production [45]. We found that *gbuA*, encoding agmatinase, converting agmatine to putrescine, was up-regulated at 8°C while expression of *gabT*, *gabD* and homologs of *puuA*, *puuB* and *puuC* were down-regulated, suggesting a reduction of putrescine degradation in cold conditions. Additionally, *algZ*, the alginate biosynthesis activator, and *algD*, a gene encoding a GDP-mannose dehydrogenase critical for alginate biosynthesis [46], were up-regulated in cold conditions. Alginate production in *P*. *extremaustralis* has been experimentally verified [10] and the induction of *algZ* could represent an early event in alginate production in cold conditions.

At low temperatures down-regulation of iron-related proteins could contribute to alleviating the oxidative stress produced by iron during the Fenton reaction [42]. In *P*. *extremaustralis*, transcriptome analysis showed that genes encoding iron-related proteins, including those involved in iron uptake and iron containing proteins were down-regulated, with the exception of bacterioferritine, which was up-regulated. Iron constitutes a key component of several TCA and aerobic respiratory chain proteins such as aconitase, citrate synthase and cytochromes and its expression is repressed in low iron environments [47]. In line with these and some observations made in *P*. *haloplanktis* and *P*. *putida* KT2440, in *P*. *extremaustralis* genes involved in TCA and respiratory cytochromes were down-regulated at cold conditions [24,42]

Therefore utilization of other pathways for growth became necessary due to the repression of genes related to main metabolic pathways observed at low temperatures. We found that genes involved in ethanol oxidation, *exaA*, *exaB*, *exaC* and *erbR*, were up-regulated in cold conditions. In *P*. *aeruginosa*, aerobic oxidation using ethanol as exogenous carbon source involving the *exaA*, *exaB* and *exaC* genes as well as the PQQ coenzyme as a prosthetic group for the *exaA* product has been described [6]. The regulatory network in *P*. *aeruginosa* includes at least seven genes, in which the different components act in a hierarchical manner [48]. The two-component regulatory system EraSR (former ExaDE) controls the transcription of the *exaA* gene [6]. This two-component system is positively regulated by the response regulator, ErbR, and expression of the *exaC* gene was reported up-regulated at 22°C relative to the expression at 37°C in *P*. *aeruginosa* [49]. We further observed that ethanol dehydrogenase activity was present at both temperatures and was impaired in the *pqqB* mutant strain, but could be partially restored in a trans-complemented strain. It is suggested here that the ethanol oxidation pathway is relevant for energy production at low temperatures, even in absence of exogenous ethanol. This suggestion is supported by the following 1) *exaA1 and pqqB* mutant strains are unable to grow at low temperatures and 2) the presence of ethanol detected by gas chromatography in LB cultures supplemented with sodium octanoate grown in cold conditions. The PQQ-dependent alcohol dehydrogenase transfers reducing equivalents directly to the cytochrome c550 component in the periplasm and constitute a short respiratory chain probably along with cytochrome c oxidase [6]. This pathway leads to a reduced-energy situation [50] in comparison with the main respiratory chain; however it becomes essential in cold conditions where TCA and cytochrome coding genes were repressed.

Ethanol production has been studied in *P*. *aeruginosa* under anaerobic conditions [5,51], where it was found during pyruvate fermentation several metabolites and also ethanol production in cultures without agitation. Although ethanol may produce alterations in the membrane and cause oxidative stress, it was reported in *P*. *putida* KT2440, carrying the *adhB* gene for ethanol production in high yield, that endogenous production of ethanol did not affect its survival even in maximum production [52]. Ethanol production in *P*. *extremaustralis* cultures grown in presence of octanoate was detected in this work by GC and is suggested here to imply that ethanol oxidation may be an alternative metabolic route in low temperature conditions. In analogy, in *P*. *putida* KT2440 the 2-methylcitrate pathway, a secondary metabolism, is activated at the transcriptional level at low temperatures [24], and it is suggested to be important as an alternative pathway. In line with this report we found that the mutation of *exaA1* in *P*. *putida* KT2440 did not affect the growth at low temperatures (data not shown), suggesting that the alternative pathways used can be different in closely related bacteria.

In summary, additional to the display of the complete profile of *P*. *extremaustralis* under low temperature, RNA-seq analysis along with traditional bacterial genetic strategies have resulted in the unexpected identification of the ethanol oxidation pathway as essential for cold growth in this bacterium. The relevance of the ethanol oxidation pathway is probably related to the ability of *P*. *extremaustralis* to use it as an alternative route for energy generation in a scenario where genes related with iron uptake, TCA and several cytochromes were repressed, probably in order to avoid ROS production during cold growth. These observations suggest that activation of unusual metabolic routes could be an important component of the overall fitness of the cellular metabolic machinery under different physico-chemical conditions, particularly for bacterial adaptability to stressful environments.

## Supporting Information

S1 Fig Dot blot representing normalized count for each RNA-seq replicate. Spearman correlation coefficient is shown for each treatment. Replicates with no expression (value of cero) are not shown.
(PDF)

S2 Fig Absorption spectrum of leuco and shift base forms of p-rosaniline at 548nm and 420 nm. (PDF)

S3 Fig Quantitative Real Time PCR: total RNA was extracted from wild type strain cultures grown at 8°C and 30°C and expression of 5 selected genes between both conditions was comparatively analyzed. Values represent the mean ± SD of three independent experiments.
(PDF)

S4 Fig GC chromatograms obtained from the analysis of culture supernatants of *P*. *extremaustralis* grown at 8°C. A. Control LB medium. B. Control LB medium plus pure ethanol. C. Supernatant of *P*. *extremaustralis*.
(DOC)

S1 Table Down-regulated genes under cold conditions in *P*. *extremaustralis*. All genes presented P≤0.05 and Q≤0.05 (Rockhopper software).
(DOC)

S2 Table Up-regulated genes under cold conditions in *P*. *extremaustralis*. All genes presented P≤0.05 and Q≤0.05. (Rockhopper software).
(DOC)

S3 Table GO categories in differentially expressed genes. (DOC)
