# Supplementary material for: Novel Essential Role of Ethanol Oxidation Genes at Low Temperature Revealed by Transcriptome Analysis in the Antarctic Bacterium Pseudomonas extremaustralis
Source: PLoS One. 2015 Dec 15;10(12):e0145353. doi: 10.1371/journal.pone.0145353 (PMC4686015; doi:10.1371/journal.pone.0145353)
Supplement: S1 Fig — Spearman correlation coefficient is shown for each treatment. Replicates with no expression (value of cero) are not shown. (PDF) [file pone.0145353.s001.pdf]

30ºC

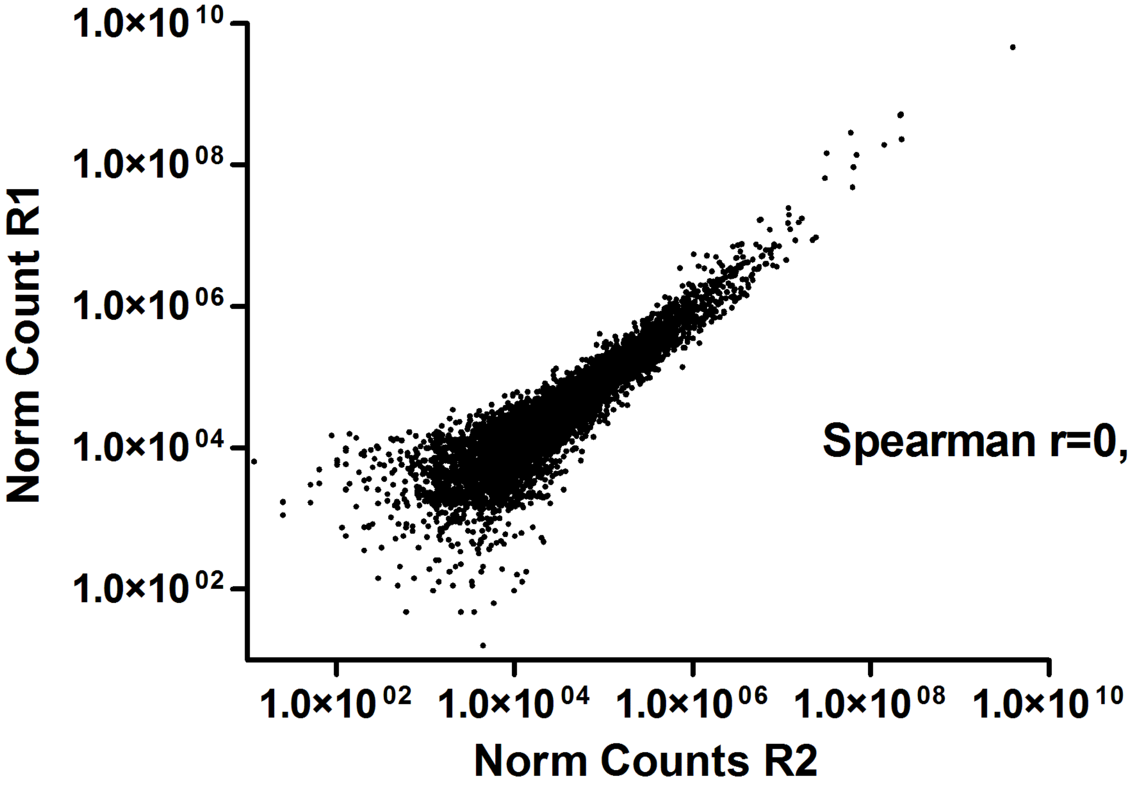

8ºC

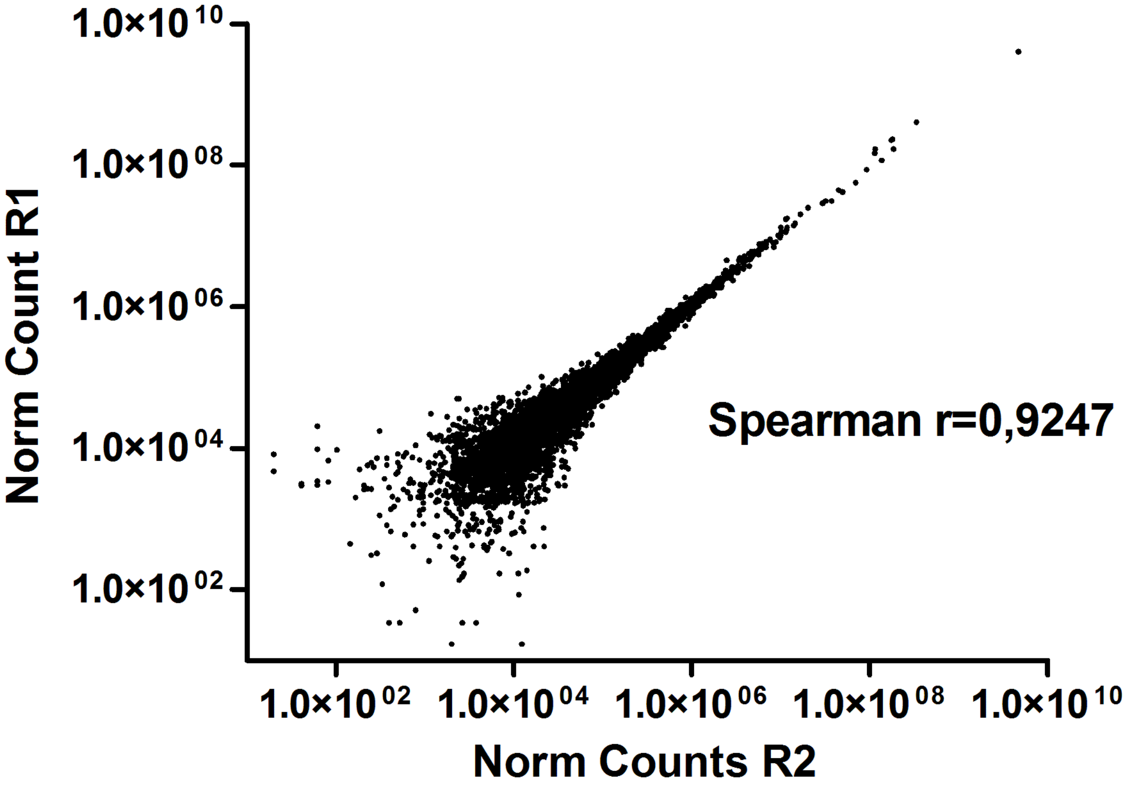

Dot blot representing normalized count for each replicate. Spearman correlation coefficient is shown for each treatment. Replicates with no expression (value of cero) are not shown
