# Supplementary material for: Novel Essential Role of Ethanol Oxidation Genes at Low Temperature Revealed by Transcriptome Analysis in the Antarctic Bacterium Pseudomonas extremaustralis
Source: PLoS One. 2015 Dec 15;10(12):e0145353. doi: 10.1371/journal.pone.0145353 (PMC4686015; doi:10.1371/journal.pone.0145353)

Absortion spectra of p-rosaniline on leuco or shift base form

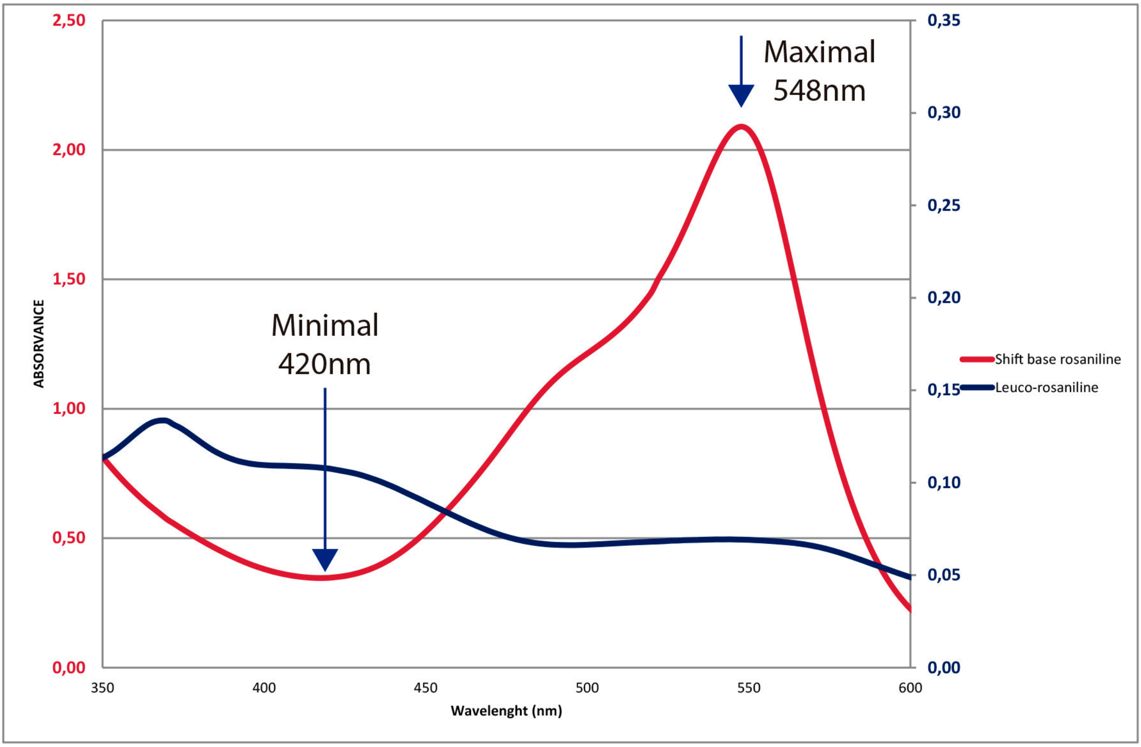

Please note the prescence of double axis coloured in the same way as the corresponding curves

Example of absortion spectra of WT and *pqqB-* strains

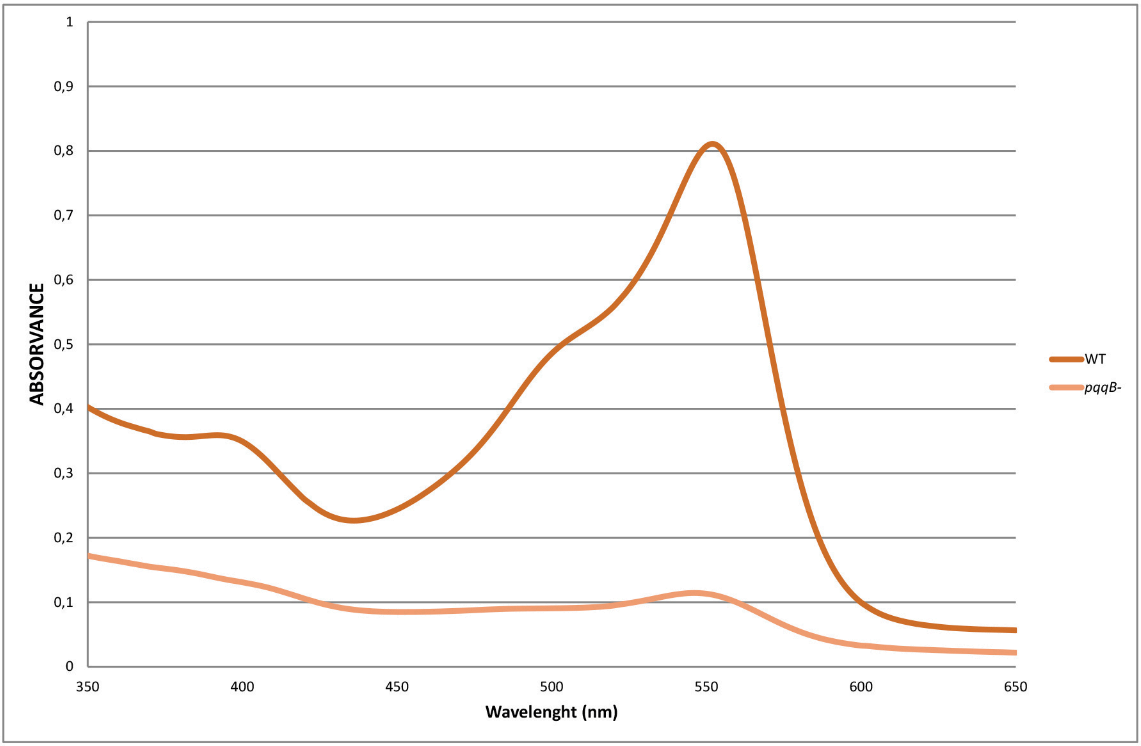

Supplement: S2 Fig — (PDF) [file pone.0145353.s002.pdf]
