# Supplementary figures and images for: Novel Essential Role of Ethanol Oxidation Genes at Low Temperature Revealed by Transcriptome Analysis in the Antarctic Bacterium Pseudomonas extremaustralis
Source: PLoS One. 2015 Dec 15;10(12):e0145353. doi: 10.1371/journal.pone.0145353 (PMC4686015; doi:10.1371/journal.pone.0145353)

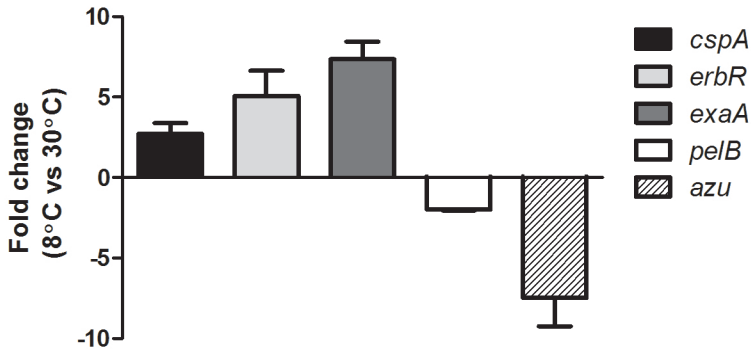

Supplement: S3 Fig — Values represent the mean ± SD of three independent experiments. (PDF) [file pone.0145353.s003.pdf]
