# Supplementary material for: Novel Essential Role of Ethanol Oxidation Genes at Low Temperature Revealed by Transcriptome Analysis in the Antarctic Bacterium Pseudomonas extremaustralis
Source: PLoS One. 2015 Dec 15;10(12):e0145353. doi: 10.1371/journal.pone.0145353 (PMC4686015; doi:10.1371/journal.pone.0145353)
Supplement: S4 Fig — A. Control LB medium. B. Control LB medium plus pure ethanol. C. Supernatant of P. extremaustralis. (DOC) [file pone.0145353.s004.doc]

A)

Fig. S4: Ethanol detection in *P.extremaustralis* grown at low temperatures during 7 days in LB+ sodium octanoate. A) LB control. B) LB+ethanol control. C) *P.extremaustralis* supernatant.

Ethanol (Retention time)

B)

C)

Ethanol in *P.extremaustralis* supernatant

(Retention time)
