# Supplementary material for: Novel Essential Role of Ethanol Oxidation Genes at Low Temperature Revealed by Transcriptome Analysis in the Antarctic Bacterium Pseudomonas extremaustralis
Source: PLoS One. 2015 Dec 15;10(12):e0145353. doi: 10.1371/journal.pone.0145353 (PMC4686015; doi:10.1371/journal.pone.0145353)
Supplement: S1 Table — All genes presented P≤0.05 and Q≤0.05 (Rockhopper software). (DOC) [file pone.0145353.s005.doc]

**S1 Table. Down-regulated genes under cold conditions in *P. extremaustralis*. All genes presented P≤0.05 and Q≤0.05 (Rockhopper software)**

| **Gene** | **Function** | **Category** | **Locus tag** | **Fold change** |
| --- | --- | --- | --- | --- |
|  | Putative large exoprotein involved in adhesion of ShlA/HecA/FhaA family | Adhesion and secretion | PE143B_0103560 | -2.8 |
|  | T1SS secreted agglutinin (RTX) | Adhesion and secretion | PE143B_0103925 | -3.4 |
| *hylD* | HlyD family secretion protein | Adhesion and secretion | PE143B_0111365 | -6.9 |
| *yajC* | Preprotein translocase subunit YajC | Adhesion and secretion | PE143B_0115215 | -2.2 |
| *hvnA* | NAD glycohydrolase. halovibrin | Adhesion and secretion | PE143B_0116905 | -6.0 |
| *yidC* | Inner membrane protein translocase component YidC | Adhesion and secretion | PE143B_0118535 | -2.2 |
|  | Large extracellular alpha-helical protein | Adhesion and secretion | PE143B_0118635 | -3.9 |
|  | Predicted secretion system X protein GspG-like | Adhesion and secretion | PE143B_0119160 | -37.8 |
| *secA* | Protein export cytoplasm protein SecA | Adhesion and secretion | PE143B_0119935 | -2.5 |
| *pto* | type III effector HopPmaJ(Pto) | Adhesion and secretion | PE143B_0120510 | -3.1 |
|  | HlyD family secretion protein | Adhesion and secretion | PE143B_0129395 | -3.7 |
|  | Agglutination protein | Adhesion and secretion | PE143B_0129405 | -4.8 |
|  |  |  |  |  |
|  | Gamma-glutamyl-putrescine oxidase | Arginine metabolism | PE143B_0103820 | -9.1 |
|  | Gamma-glutamyl-putrescine synthetase | Arginine metabolism | PE143B_0103830 | -7.4 |
|  | Gamma-glutamyl-GABA hydrolase | Arginine metabolism | PE143B_0103835 | -12.9 |
|  | Lysine-arginine-ornithine-binding periplasmic protein precursor | Arginine metabolism | PE143B_0104380 | -2.6 |
| *argG* | Argininosuccinate synthase | Arginine metabolism | PE143B_0109535 | -2.7 |
|  | Putrescine ABC transporter putrescine-binding protein PotF | Arginine metabolism | PE143B_0111180 | -2.9 |
| *argA* | N-acetylglutamate synthase | Arginine metabolism | PE143B_0111300 | -3.9 |
| *arcC* | Ornithine carbamoyltransferase | Arginine metabolism | PE143B_0120165 | -45.6 |
| *arcA* | Arginine deiminase | Arginine metabolism | PE143B_0120170 | -20.5 |
| *arcD* | Arginine/ornithine antiporter ArcD | Arginine metabolism | PE143B_0120175 | -12.3 |
|  | Succinylglutamate desuccinylase | Arginine metabolism | PE143B_0122425 | -3.2 |
| *astD* | Succinylglutamic semialdehyde dehydrogenase | Arginine metabolism | PE143B_0122440 | -5.5 |
|  | Arginine N-succinyltransferase. alpha subunit | Arginine metabolism | PE143B_0122450 | -3.6 |
| *aruC* | Succinylornithine transaminase | Arginine metabolism | PE143B_0122455 | -4.2 |
| *aotP* | Arginine/ornithine ABC transporter. ATP-binding protein AotP | Arginine metabolism | PE143B_0122470 | -4.6 |
| *aotJ* | Arginine/ornithine ABC transporter. periplasmic arginine/ornithine binding protein | Arginine metabolism | PE143B_0122490 | -2.9 |
| *argB* | Acetylglutamate kinase | Arginine metabolism | PE143B_0124540 | -3.3 |
| *argH* | Argininosuccinate lyase | Arginine metabolism | PE143B_0125840 | -2.9 |
|  |  |  |  |  |
|  | Rare lipoprotein A precursor | Cell wall and membrane | PE143B_0100580 | -2.5 |
|  | N-acetylmannosaminyltransferase | Cell wall and membrane | PE143B_0101410 | -5.3 |
| *waaL* | Surface polymer ligase WaaL | Cell wall and membrane | PE143B_0101420 | -9.1 |
| *glmM* | Phosphoglucosamine mutase | Cell wall and membrane | PE143B_0101845 | -2.2 |
|  | Multimodular transpeptidase-transglycosylase | Cell wall and membrane | PE143B_0101965 | -3.6 |
|  | Penicillin amidase family protein | Cell wall and membrane | PE143B_0102165 | -18.7 |
|  | Multimodular transpeptidase-transglycosylase | Cell wall and membrane | PE143B_0104570 | -2.6 |
|  | Carbamoyltransferase in large core OS assembly cluster | Cell wall and membrane | PE143B_0104920 | -2.7 |
| *amiB* | N-acetylmuramoyl-L-alanine amidase | Cell wall and membrane | PE143B_0105145 | -2.9 |
|  | Membrane-bound lytic murein transglycosylase D precursor | Cell wall and membrane | PE143B_0112840 | -2.9 |
|  | Transglycosylase. Slt family | Cell wall and membrane | PE143B_0115395 | -3.5 |
| *pagL* | Lipid A 3-O-deacylase | Cell wall and membrane | PE143B_0117290 | -4.4 |
| *mraY* | Phospho-N-acetylmuramoyl-pentapeptide-transferase | Cell wall and membrane | PE143B_0117605 | -2.6 |
| *htrB* | Lipid A biosynthesis lauroyl acyltransferase | Cell wall and membrane | PE143B_0118465 | -2.8 |
| *glmU* | N-acetylglucosamine-1-phosphate uridyltransferase/ Glucosamine-1-phosphate N-acetyltransferase | Cell wall and membrane | PE143B_0118610 | -4.1 |
| *wzx* | O-antigen flippase Wzx | Cell wall and membrane | PE143B_0121925 | -2.3 |
|  | Membrane-associated phospholipid phosphatase | Cell wall and membrane | PE143B_0125395 | -18.4 |
|  | Pirin | Cell wall and membrane | PE143B_0129900 | -21.2 |
|  |  |  |  |  |
| *ftsH* | Cell division protein FtsH | Cellular Division | PE143B_0101775 | -2.3 |
| *ftsN* | Cell division protein FtsN | Cellular Division | PE143B_0104535 | -4.1 |
| *ftsX* | Cell division protein FtsX | Cellular Division | PE143B_0110885 | -2.2 |
| *mraZ* | Cell division protein MraZ | Cellular Division | PE143B_0117635 | -2.7 |
| *mreB* | Rod shape-determining protein MreB | Cellular Division | PE143B_0128290 | -2.7 |
|  |  |  |  |  |
| *grpE* | Heat shock protein GrpE | Chaperone | PE143B_0101725 | -4.9 |
| *dnaK* | Chaperone protein DnaK | Chaperone | PE143B_0101730 | -4.6 |
| *dnaK* | Chaperone protein DnaK | Chaperone | PE143B_0101735 | -2.6 |
| *dnaJ* | Chaperone protein DnaJ | Chaperone | PE143B_0101385 | -8.5 |
| *secB* | Protein export cytoplasm chaperone protein SecB | Chaperone | PE143B_0104220 | -3.2 |
| *cbpA* | DnaJ-class molecular chaperone CbpA | Chaperone | PE143B_0105440 | -2.7 |
| *htrA* | HtrA protease/chaperone protein | Chaperone | PE143B_0107140 | -2.6 |
| *htpG* | Chaperone protein HtpG | Chaperone | PE143B_0113980 | -7.3 |
| *htpX* | Protease HtpX | Chaperone | PE143B_0114340 | -4.3 |
| *groES* | Heat shock protein 60 family co-chaperone GroES | Chaperone | PE143B_0115645 | -5.6 |
| *groEL* | Heat shock protein 60 family chaperone GroEL | Chaperone | PE143B_0115650 | -6.2 |
| *clpB* | ClpB protein | Chaperone | PE143B_0117090 | -5.0 |
| *slpA* | FKBP-type peptidyl-prolyl cis-trans isomerase slpA | Chaperone | PE143B_0117150 | -3.2 |
| *cspC* | Cold shock protein CspC | Chaperone | PE143B_0119990 | -5.5 |
|  | Cold-shock protein | Chaperone | PE143B_0120120 | -44.2 |
| *hslO* | 33 kDa heat shock chaperonin | Chaperone | PE143B_0125400 | -4.0 |
| *ibpA (like)* | Heat shock protein Hsp20 | Chaperone | PE143B_0127215 | -94.0 |
|  |  |  |  |  |
|  | Putative oxidoreductase YncB | Cythochromes, quinone and oxidoreductase protein | PE143B_0100190 | -3.3 |
| *iorA* | Isoquinoline 1-oxidoreductase alpha subunit | Cythochromes, quinone and oxidoreductase protein | PE143B_0100195 | -10.0 |
|  | Putative diheme cytochrome c-553 | Cythochromes, quinone and oxidoreductase protein | PE143B_0100205 | -5.9 |
|  | Electron transfer flavoprotein. beta subunit | Cythochromes, quinone and oxidoreductase protein | PE143B_0100365 | Not present at 8°C |
|  | Oxidoreductase | Cythochromes, quinone and oxidoreductase protein | PE143B_0101290 | -5.3 |
| *azu* | Azurin | Cythochromes, quinone and oxidoreductase protein | PE143B_0105285 | -5.6 |
|  | L-pipecolate oxidase | Cythochromes, quinone and oxidoreductase protein | PE143B_0105320 | -3.3 |
| *fpr* | Ferredoxin--NADP(+) reductase | Cythochromes, quinone and oxidoreductase protein | PE143B_0105900 | -3.4 |
|  | NADH:flavin oxidoreductase | Cythochromes, quinone and oxidoreductase protein | PE143B_0106730 | -8.7 |
|  | Electron transfer flavoprotein-ubiquinone oxidoreductase | Cythochromes, quinone and oxidoreductase protein | PE143B_0107550 | -2.2 |
| *etfA* | Electron transfer flavoprotein. alpha subunit | Cythochromes, quinone and oxidoreductase protein | PE143B_0107560 | -2.1 |
| *cumA* | Multicopper oxidase | Cythochromes, quinone and oxidoreductase protein | PE143B_0109175 | -3.5 |
|  | Cytochrome B561 | Cythochromes, quinone and oxidoreductase protein | PE143B_0110615 | -6.0 |
|  | D-2-hydroxyglutarate dehydrogenase | Cythochromes, quinone and oxidoreductase protein | PE143B_0111105 | -3.5 |
| *gpsA* | Glycerol-3-phosphate dehydrogenase [NAD(P)+] | Cythochromes, quinone and oxidoreductase protein | PE143B_0113935 | -3.0 |
|  | FAD/FMN-containing dehydrogenases | Cythochromes, quinone and oxidoreductase protein | PE143B_0117050 | -6.7 |
|  | Cytochrome O ubiquinol oxidase subunit II | Cythochromes, quinone and oxidoreductase protein | PE143B_0120630 | -2.3 |
|  | Cytochrome O ubiquinol oxidase subunit I | Cythochromes, quinone and oxidoreductase protein | PE143B_0120635 | -3.3 |
|  | Cytochrome O ubiquinol oxidase subunit | Cythochromes, quinone and oxidoreductase protein | PE143B_0120640 | -2.9 |
|  | Cytochrome O ubiquinol oxidase subunit IV | Cythochromes, quinone and oxidoreductase protein | PE143B_0120645 | -3.5 |
|  | Oxidoreductase. short chain dehydrogenase/reductase family | Cythochromes, quinone and oxidoreductase protein | PE143B_0121845 | -4.1 |
|  | Quinate/shikimate dehydrogenase [Pyrroloquinoline-quinone] | Cythochromes, quinone and oxidoreductase protein | PE143B_0122335 | -7.9 |
| *vanB* | Flavodoxin reductases (ferredoxin-NADPH reductases) family 1; Vanillate O-demethylase oxidoreductase | Cythochromes, quinone and oxidoreductase protein | PE143B_0122765 | -2.2 |
| *cc4* | Cytochrome c4 | Cythochromes, quinone and oxidoreductase protein | PE143B_0123015 | -2.3 |
| *gcdH* | Glutaryl-CoA dehydrogenase | Cythochromes, quinone and oxidoreductase protein | PE143B_0123170 | -4.6 |
| *rubB (alkT)* | Rubredoxin-NAD(+) reductase | Cythochromes, quinone and oxidoreductase protein | PE143B_0124430 | -3.8 |
| *petB* | Ubiquinol--cytochrome c reductase. cytochrome B subunit | Cythochromes, quinone and oxidoreductase protein | PE143B_0130190 | -2.4 |
|  |  |  |  |  |
|  | Aldehyde dehydrogenase | Ethanol metabolism | PE143B_0100250 | -15.7 |
|  | Alcohol dehydrogenase | Ethanol metabolism | PE143B_0100280 | -10.4 |
|  | Short-chain alcohol dehydrogenase protein | Ethanol metabolism | PE143B_0121820 | -2.4 |
|  |  |  |  |  |
|  | Sugar transferase protein | Exopolysaccharide | PE143B_0101375 | -18.9 |
| *galE* | UDP-glucose 4-epimerase | Exopolysaccharide | PE143B_0101385 | -17.5 |
|  | Polysaccharide deacetylase | Exopolysaccharide | PE143B_0114940 | -8.4 |
|  | Alginate lyase precursor | Exopolysaccharide | PE143B_0118910 | -9.4 |
| *gmd* | GDP-mannose 4.6-dehydratase | Exopolysaccharide | PE143B_0121965 | -2.4 |
| *cpsB* | Mannose-1-phosphate guanylyltransferase (GDP) | Exopolysaccharide | PE143B_0121975 | -2.1 |
| *galE* | UDP-glucose 4-epimerase | Exopolysaccharide | PE143B_0121985 | -3.1 |
| *galE* | UDP-glucose 4-epimerase | Exopolysaccharide | PE143B_0125540 | -23.8 |
| *pelA* | Extracellular Matrix protein PelA | Exopolysaccharide | PE143B_0125545 |  |
| *pelB* | Extracellular Matrix protein PelB | Exopolysaccharide | PE143B_0125550 | -39.9 |
| *pelC* | Extracellular Matrix protein PelC | Exopolysaccharide | PE143B_0125555 | -13.5 |
| *pelD* | Extracellular Matrix protein PelD | Exopolysaccharide | PE143B_0125560 | -32.4 |
| *pelE* | Extracellular Matrix protein PelE | Exopolysaccharide | PE143B_0125565 | -38.8 |
| *pelf* | Extracellular matrix protein PelF | Exopolysaccharide | PE143B_0125570 | -21.5 |
| *pelG* | Extracellular Matrix protein PelG | Exopolysaccharide | PE143B_0125575 | -43.3 |
|  |  |  |  |  |
| *atoB* | Acetyl-CoA acetyltransferase/Beta-ketoadipyl CoA thiolase | Fatty acids metabolism | PE143B_0100355 | -48.8 |
|  | Acetyl-CoA acetyltransferase | Fatty acids metabolism | PE143B_0101300 | -23.6 |
| *pssA-1* | CDP-diacylglycerol--serine O-phosphatidyltransferase | Fatty acids metabolism | PE143B_0101995 | -3.4 |
| *fabI* | Enoyl-[acyl-carrier-protein] reductase [FMN] | Fatty acids metabolism | PE143B_0102425 | -7.2 |
|  | 3-hydroxyisobutyryl-CoA hydrolase | Fatty acids metabolism | PE143B_0102695 | -5.2 |
|  | Acyl carrier protein | Fatty acids metabolism | PE143B_0104730 | -8.3 |
|  | Long-chain fatty acid transport protein | Fatty acids metabolism | PE143B_0108495 | -2.4 |
|  | Long-chain-fatty-acid--CoA ligase | Fatty acids metabolism | PE143B_0110335 | -5.2 |
| *fabB* | 3-oxoacyl-[acyl-carrier-protein] synthase. KASI | Fatty acids metabolism | PE143B_0113960 | -2.6 |
| *coaBC* | Phosphopantothenoylcysteine synthetase/decarboxylase | Fatty acids metabolism | PE143B_0114755 | -11.3 |
| *cfa* | Cyclopropane-fatty-acyl-phospholipid synthase | Fatty acids metabolism | PE143B_0118685 | -4.4 |
| *rhlA* | RhlA. 3-(3-hydroxyalkanoyloxy)alkanoic acids (HAAs) synthase | Fatty acids metabolism | PE143B_0118960 | -17.3 |
|  | Acyl-CoA dehydrogenase | Fatty acids metabolism | PE143B_0120895 | -2.9 |
| *plsX* | Phosphate:acyl-ACP acyltransferase PlsX | Fatty acids metabolism | PE143B_0122185 | -7.1 |
|  | Acetyl-coenzyme A synthetase | Fatty acids metabolism | PE143B_0122495 | -5.5 |
|  | Acetyl-CoA hydrolase | Fatty acids metabolism | PE143B_0123145 | -6.1 |
|  | Acetyltransferase | Fatty acids metabolism | PE143B_0124270 | #¡DIV/0! |
|  | Acyl-CoA dehydrogenase | Fatty acids metabolism | PE143B_0127635 | -3.1 |
|  | Protein in KDO2-Lipid A biosynthesis cluster | Fatty acids metabolism | PE143B_0128970 | -7.1 |
|  |  |  |  |  |
| *gpmI* | 2.3-bisphosphoglycerate-independent phosphoglycerate mutase | Glycolysis and pentose cycle | PE143B_0104205 | -3.7 |
| *tme* | NADP-dependent malic enzyme | Glycolysis and pentose cycle | PE143B_0104565 | -4.4 |
| *eno* | Enolase | Glycolysis and pentose cycle | PE143B_0106340 | -2.3 |
|  | NADPH-dependent glyceraldehyde-3-phosphate dehydrogenase | Glycolysis and pentose cycle | PE143B_0107545 | -4.7 |
| *glpD* | Aerobic glycerol-3-phosphate dehydrogenase | Glycolysis and pentose cycle | PE143B_0109460 | -4.7 |
| *glpK* | Glycerol kinase | Glycolysis and pentose cycle | PE143B_0109465 | -3.0 |
| *gapA* | NAD-dependent glyceraldehyde-3-phosphate dehydrogenase | Glycolysis and pentose cycle | PE143B_0115740 | -2.8 |
| *gabD* | Glutarate-semialdehyde dehydrogenase/ Succinate-semialdehyde dehydrogenase [NADP+] | Glycolysis and pentose cycle | PE143B_0116050 | -3.1 |
| *mqo* | Malate:quinone oxidoreductase | Glycolysis and pentose cycle | PE143B_0117835 | -3.2 |
|  | Transketolase | Glycolysis and pentose cycle | PE143B_0118875 | -3.2 |
| *pgk* | Phosphoglycerate kinase | Glycolysis and pentose cycle | PE143B_0118885 | -4.0 |
| *fba* | Fructose-bisphosphate aldolase class II | Glycolysis and pentose cycle | PE143B_0118900 | -3.1 |
| *glcB* | Malate synthase G | Glycolysis and pentose cycle | PE143B_0120825 | -3.3 |
| *rbsK* | Ribokinase | Glycolysis and pentose cycle | PE143B_0124905 | -5.6 |
| *pckA* | Phosphoenolpyruvate carboxykinase | Glycolysis and pentose cycle | PE143B_0125405 | -4.9 |
|  |  |  |  |  |
|  | Hypothetical protein | HP | PE143B_0100100 | -3.5 |
|  | Hypothetical protein | HP | PE143B_0100115 | -2.8 |
|  | Hypothetical protein | HP | PE143B_0100120 | -2.7 |
|  | Hypothetical protein | HP | PE143B_0100155 | -4.4 |
|  | Hypothetical protein | HP | PE143B_0100160 | -2.5 |
|  | Hypothetical protein | HP | PE143B_0100380 | -45.6 |
|  | Hypothetical protein | HP | PE143B_0100515 | -17.3 |
|  | Hypothetical protein | HP | PE143B_0100795 | -3.1 |
|  | Hypothetical protein | HP | PE143B_0100870 | -4.2 |
|  | Hypothetical protein | HP | PE143B_0100875 | -5.9 |
|  | Hypothetical protein | HP | PE143B_0100935 | -11.3 |
|  | Hypothetical protein | HP | PE143B_0101180 | -22.0 |
|  | Hypothetical protein | HP | PE143B_0101315 | -3.4 |
|  | Hypothetical protein | HP | PE143B_0101320 | -9.4 |
|  | Hypothetical protein | HP | PE143B_0101345 | -42.5 |
|  | Hypothetical protein | HP | PE143B_0101365 | -8.7 |
|  | Hypothetical protein | HP | PE143B_0101385 | -8.5 |
|  | Hypothetical protein | HP | PE143B_0101385 | -4.6 |
|  | Hypothetical protein | HP | PE143B_0101645 | -4.0 |
|  | Hypothetical protein | HP | PE143B_0101645 | -2.6 |
|  | Hypothetical protein | HP | PE143B_0101645 | -3.0 |
|  | Hypothetical protein | HP | PE143B_0101660 | -3.4 |
|  | Hypothetical protein | HP | PE143B_0101965 | -3.2 |
|  | Hypothetical protein | HP | PE143B_0101970 | -2.7 |
|  | Hypothetical protein | HP | PE143B_0101975 | -2.4 |
|  | Hypothetical protein | HP | PE143B_0102035 | -16.5 |
|  | Hypothetical protein | HP | PE143B_0103995 | -4.1 |
|  | Hypothetical protein | HP | PE143B_0104055 | -3.3 |
|  | Hypothetical protein | HP | PE143B_0104270 | -4.0 |
|  | Hypothetical protein | HP | PE143B_0105080 | -2.3 |
|  | Hypothetical protein | HP | PE143B_0105615 | -4.4 |
|  | Hypothetical protein | HP | PE143B_0105850 | -3.1 |
|  | Hypothetical protein | HP | PE143B_0105870 | -2.9 |
|  | Hypothetical protein | HP | PE143B_0106280 | -2.9 |
|  | Hypothetical protein | HP | PE143B_0106510 | -2.8 |
|  | Hypothetical protein | HP | PE143B_0106625 | -10.9 |
|  | Hypothetical protein | HP | PE143B_0106635 | -5.3 |
|  | Hypothetical protein | HP | PE143B_0107200 | -15.7 |
|  | Hypothetical protein | HP | PE143B_0108375 | -3.9 |
|  | Hypothetical protein | HP | PE143B_0108380 | -2.7 |
|  | Hypothetical protein | HP | PE143B_0109545 | -4.6 |
|  | Hypothetical protein | HP | PE143B_0109720 | -42.5 |
|  | Hypothetical protein | HP | PE143B_0110595 | -33.1 |
|  | Hypothetical protein | HP | PE143B_0110610 | -2.5 |
|  | Hypothetical protein | HP | PE143B_0110715 | -11.0 |
|  | Hypothetical protein | HP | PE143B_0111115 | -4.8 |
|  | Hypothetical protein | HP | PE143B_0112260 | -21.0 |
|  | Hypothetical protein | HP | PE143B_0112605 | -18.4 |
|  | Hypothetical protein | HP | PE143B_0113455 | -18.9 |
|  | Hypothetical protein | HP | PE143B_0113725 | -4.5 |
|  | Hypothetical protein | HP | PE143B_0114470 | -2.9 |
|  | Hypothetical protein | HP | PE143B_0114660 | -3.4 |
|  | Hypothetical protein | HP | PE143B_0114665 | -5.6 |
|  | Hypothetical protein | HP | PE143B_0115180 | -5.3 |
|  | Hypothetical protein | HP | PE143B_0115340 | -5.7 |
|  | Hypothetical protein | HP | PE143B_0115445 | -8.1 |
|  | Hypothetical protein | HP | PE143B_0115250 | -15.0 |
|  | Hypothetical protein | HP | PE143B_0115830 | -4.6 |
|  | Hypothetical protein | HP | PE143B_0116420 | -13.4 |
|  | Hypothetical protein | HP | PE143B_0116915 | -9.6 |
|  | Hypothetical protein | HP | PE143B_0117210 | -36.2 |
|  | Hypothetical protein | HP | PE143B_0117400 | -6.2 |
|  | Hypothetical protein | HP | PE143B_0117665 | -2.8 |
|  | Hypothetical protein | HP | PE143B_0117700 | -2.1 |
|  | Hypothetical protein | HP | PE143B_0117815 | -7.6 |
|  | Hypothetical protein | HP | PE143B_0117890 | -3.3 |
|  | Hypothetical protein | HP | PE143B_0118210 | -3.4 |
|  | Hypothetical protein | HP | PE143B_0118625 | -10.7 |
|  | Hypothetical protein | HP | PE143B_0118770 | -2.6 |
|  | Hypothetical protein | HP | PE143B_0119020 | -2.7 |
|  | Hypothetical protein | HP | PE143B_0119475 | -9.9 |
|  | Hypothetical protein | HP | PE143B_0119640 | -5.8 |
|  | Hypothetical protein | HP | PE143B_0119965 | -8.4 |
|  | Hypothetical protein | HP | PE143B_0120020 | -4.1 |
|  | Hypothetical protein | HP | PE143B_0120110 | -4.2 |
|  | Hypothetical protein | HP | PE143B_0120660 | -3.5 |
|  | Hypothetical protein | HP | PE143B_0120885 | -4.0 |
|  | Hypothetical protein | HP | PE143B_0121575 | -11.2 |
|  | Hypothetical protein | HP | PE143B_0121955 | -3.6 |
|  | Hypothetical protein | HP | PE143B_0122200 | -15.7 |
|  | Hypothetical protein | HP | PE143B_0122645 | -2.7 |
|  | Hypothetical protein | HP | PE143B_0122760 | -2.1 |
|  | Hypothetical protein | HP | PE143B_0122770 | -2.3 |
|  | Hypothetical protein | HP | PE143B_0122845 | -7.9 |
|  | Hypothetical protein | HP | PE143B_0122995 | -3.2 |
|  | Hypothetical protein | HP | PE143B_0123120 | -5.8 |
|  | Hypothetical protein | HP | PE143B_0123125 | -22.9 |
|  | Hypothetical protein | HP | PE143B_0123140 | -5.9 |
|  | Hypothetical protein | HP | PE143B_0123200 | -2.7 |
|  | Hypothetical protein | HP | PE143B_0123205 | -6.0 |
|  | Hypothetical protein | HP | PE143B_0123375 | -9.2 |
|  | Hypothetical protein | HP | PE143B_0123600 | -12.8 |
|  | Hypothetical protein | HP | PE143B_0125125 | -69.2 |
|  | Hypothetical protein | HP | PE143B_0125140 | -13.5 |
|  | Hypothetical protein | HP | PE143B_0125530 | -14.7 |
|  | Hypothetical protein | HP | PE143B_0125765 | -4.0 |
|  | Hypothetical protein | HP | PE143B_0125770 | -3.4 |
|  | Hypothetical protein | HP | PE143B_0126525 | -11.0 |
|  | Hypothetical protein | HP | PE143B_0127425 | -8.9 |
|  | Hypothetical protein | HP | PE143B_0127465 | -2.1 |
|  | Hypothetical protein | HP | PE143B_0127475 | -2.7 |
|  | Hypothetical protein | HP | PE143B_0128030 | -5.6 |
|  | Hypothetical protein | HP | PE143B_0128035 | -18.1 |
|  | Hypothetical protein | HP | PE143B_0128185 | -13.1 |
|  | Hypothetical protein | HP | PE143B_0128215 | -4.9 |
|  | Hypothetical protein | HP | PE143B_0128225 | -8.5 |
|  | Hypothetical protein | HP | PE143B_0128235 | -3.7 |
|  | Hypothetical protein | HP | PE143B_0128515 | -4.9 |
|  | Hypothetical protein | HP | PE143B_0128625 | -2.9 |
|  | Hypothetical protein | HP | PE143B_0128700 | -3.3 |
|  | Hypothetical protein | HP | PE143B_0128780 | -2.4 |
|  | Hypothetical protein | HP | PE143B_0128790 | -2.6 |
|  | Hypothetical protein | HP | PE143B_0128805 | -2.1 |
|  | Hypothetical protein | HP | PE143B_0128860 | -15.5 |
|  | Hypothetical protein | HP |  | -18.1 |
|  | Hypothetical protein | HP | PE143B_0130490 | -44.1 |
|  | Hypothetical protein | HP | PE143B_0130495 | -12.9 |
|  | Hypothetical protein | HP | PE143B_0130515 | -32.2 |
|  | Hypothetical protein | HP | PE143B_0130520 | -37.9 |
|  | Hypothetical protein | HP | PE143B_0130540 | -6.4 |
|  | Hypothetical protein | HP | PE143B_0130545 | -26.8 |
|  | Hypothetical protein | HP | PE143B_0130570 | -67.7 |
|  | Hypothetical protein | HP | PE143B_0130765 | -138.5 |
|  | Hypothetical protein | HP | PE143B_0130920 | -26.8 |
|  | Hypothetical protein | HP | PE143B_0129270 | -18.1 |
|  |  |  |  |  |
|  | Periplasmic hemin-binding protein | Iron and pyoverdin | PE143B_0101940 | -5.7 |
| *phuU* | Hemin ABC transporter. permease protein | Iron and pyoverdin | PE143B_0101945 | -5.6 |
|  | ABC-type hemin transport system. ATPase component | Iron and pyoverdin | PE143B_0101960 | -5.0 |
| *fpvA* | Outer membrane ferripyoverdine receptor | Iron and pyoverdin | PE143B_0102060 | -40.1 |
|  | Fe2+/Zn2+ uptake regulation protein | Iron and pyoverdin | PE143B_0102160 | -143.2 |
|  | Ferric iron ABC transporter. iron-binding protein | Iron and pyoverdin | PE143B_0105200 | -7.7 |
|  | Outer membrane receptor protein. Fe transport | Iron and pyoverdin | PE143B_0105515 | -5.1 |
|  | Iron uptake protein | Iron and pyoverdin | PE143B_0109080 | -14.5 |
|  | Iron-regulated membrane protein | Iron and pyoverdin | PE143B_0109085 | -11.4 |
| *bfd* | Bacterioferritin-associated ferredoxin | Iron and pyoverdin | PE143B_0109510 | -6.2 |
|  | Non-ribosomal peptide synthetase modules. pyoverdine | Iron and pyoverdin | PE143B_0110320 | -12.2 |
| *pvdD* | Pyoverdine sidechain non-ribosomal peptide synthetase PvdD | Iron and pyoverdin | PE143B_0110325 | -12.6 |
|  | Peptide synthase | Iron and pyoverdin | PE143B_0110330 | -9.4 |
| *fpvA* | Outer membrane ferripyoverdine receptor FpvA | Iron and pyoverdin | PE143B_0110340 | -7.3 |
| *pvdE* | PvdE. pyoverdine ABC export system. fused ATPase and permease components | Iron and pyoverdin | PE143B_0110345 | -26.1 |
| *pvdF* | Pyoverdine synthetase PvdF. N5-hydroxyornithine formyltransferase | Iron and pyoverdin | PE143B_0110350 | -13.9 |
| *pvdO* | PvdO. pyoverdine responsive serine | Iron and pyoverdin | PE143B_0110360 | -11.0 |
| *pvdM* | Pyoverdin dipeptidase biosynthesis PvdM | Iron and pyoverdin | PE143B_0110365 | -49.4 |
| *pvdP* | Pyoverdine biosynthesis related protein PvdP | Iron and pyoverdin | PE143B_0110370 | -5.7 |
|  | Ferric iron ABC transporter. permease protein | Iron and pyoverdin | PE143B_0111305 | -3.3 |
|  | Ferric iron ABC transporter. iron-binding protein | Iron and pyoverdin | PE143B_0111310 | -3.5 |
|  | Outer membrane porin. coexpressed with pyoverdine biosynthesis regulon | Iron and pyoverdin | PE143B_0111810 | -8.8 |
|  | ABC transporter in pyoverdin gene cluster. periplasmic component | Iron and pyoverdin | PE143B_0115150 | -4.1 |
|  | ABC transporter in pyoverdin gene cluster. permease component | Iron and pyoverdin | PE143B_0115155 | -5.9 |
|  | Protein in pyoverdin gene cluster | Iron and pyoverdin | PE143B_0115170 | -49.6 |
|  | Protein in pyoverdin gene cluster | Iron and pyoverdin | PE143B_0115175 | -6.0 |
|  | Iron-regulated membrane protein | Iron and pyoverdin | PE143B_0115185 | -6.4 |
| *fdxA* | Ferredoxin | Iron and pyoverdin | PE143B_0115280 | -2.3 |
|  | TonB-dependent hemin . ferrichrome receptor | Iron and pyoverdin | PE143B_0115725 | -19.6 |
| *efeB* | Ferrous iron transport peroxidase EfeB | Iron and pyoverdin | PE143B_0116340 | -6.4 |
| *piuC* | Iron-uptake factor PiuC | Iron and pyoverdin | PE143B_0116920 | -5.9 |
|  | Ferrichrome-iron receptor | Iron and pyoverdin | PE143B_0116925 | -8.6 |
| *pvdA* | L-ornithine 5-monooxygenase. PvdA of pyoverdin biosynthesis | Iron and pyoverdin | PE143B_0117850 | -14.6 |
| *fpvI* | Sigma-70 factor FpvI. controling pyoverdin biosynthesis | Iron and pyoverdin | PE143B_0117855 | -9.4 |
| *macA* | Pyoverdine-specific efflux macA-like protein | Iron and pyoverdin | PE143B_0117860 | -163.7 |
|  | Pyoverdine efflux carrier and ATP binding protein | Iron and pyoverdin | PE143B_0117865 | -12.4 |
|  | Outer membrane pyoverdine eflux protein | Iron and pyoverdin | PE143B_0117870 | Not present at 8°C |
| *ctaB* | Heme O synthase. protoheme IX farnesyltransferase | Iron and pyoverdin | PE143B_0120650 | -2.7 |
| *pvdS* | Sigma factor PvdS. controling pyoverdin biosynthesis | Iron and pyoverdin | PE143B_0121275 | -25.8 |
| *pvdG* | Thioesterase PvdG involved in non-ribosomal peptide biosynthesis | Iron and pyoverdin | PE143B_0121280 | -10.7 |
| *pvdL* | Pyoverdine chromophore precursor synthetase PvdL | Iron and pyoverdin | PE143B_0121285 | -20.7 |
| *pvdH* | Pyoverdin biosynthesis protein PvdH. L-2.4-diaminobutyrate:2-oxoglutarate aminotransferase | Iron and pyoverdin | PE143B_0121320 | -18.7 |
|  | Outer membrane receptor protein. mostly Fe transport | Iron and pyoverdin | PE143B_0123135 | -27.9 |
| *tonB* | Ferric siderophore transport system. periplasmic binding protein TonB | Iron and pyoverdin | PE143B_0124470 | -4.5 |
|  | Iron-regulated protein A precursor | Iron and pyoverdin | PE143B_0125300 | -6.1 |
|  | Ferrichrome-iron receptor | Iron and pyoverdin | PE143B_0126495 | -3.7 |
|  | Iron binding protein from the HesB_IscA_SufA family | Iron and pyoverdin | PE143B_0130135 | -2.3 |
|  |  |  |  |  |
| *nn* | D-lactate dehydrogenase. Fe-S protein. FAD/FMN-containing | Miscellaneous | PE143B_0101675 | -9.4 |
| *cysC* | adenylylsulfate kinase( EC:2.7.1.25 ) | Miscellaneous | PE143B_0104975 | -8.1 |
|  | granule-associated protein | Miscellaneous | PE143B_0105765 | -2.1 |
| *thiS* | Sulfur carrier protein ThiS | Miscellaneous | PE143B_0110865 | -6.5 |
| *gloB* | Hydroxyacylglutathione hydrolase | Miscellaneous | PE143B_0112835 | -5.2 |
| *ilvE* | Cytosolic long-chain acyl-CoA thioester hydrolase family protein | Miscellaneous | PE143B_0118825 | -3.9 |
| *impB* | Phosphatase YieH | Miscellaneous | PE143B_0119750 | -8.1 |
| *fagA* | Glycosyltransferase | Miscellaneous | PE143B_0121920 | -2.6 |
|  | Glycosyl transferase. group 2 family protein | Miscellaneous | PE143B_0121980 | -2.6 |
| *yieH* | Extracellular deoxyribonuclease Dns | Miscellaneous | PE143B_0124810 | -4.3 |
|  | L-lactate dehydrogenase | Miscellaneous | PE143B_0127200 | -5.3 |
|  | Protein often clustered or fused with uracil-DNA glycosylase | Miscellaneous | PE143B_0113155 | -5.7 |
| *dns* | Uncharacterized protein ImpB | Miscellaneous | PE143B_0118310 | -11 |
| *lldD* | FagA protein | Miscellaneous | PE143B_0128340 | Not present at 8°C |
|  |  |  |  |  |
|  | Site-specific recombinase. phage integrase family | Mobile elements | PE143B_0100775 | -2.4 |
|  | Site-specific recombinase. phage integrase family | Mobile elements | PE143B_0100785 | -2.9 |
|  | Mobile element protein | Mobile elements | PE143B_0100815 | -9.0 |
|  | Mobile element protein | Mobile elements | PE143B_0100845 | -5.0 |
|  | Mobile element protein | Mobile elements | PE143B_0100860 | -5.6 |
|  | Mobile element protein | Mobile elements | PE143B_0101665 | -3.0 |
|  | Phage integrase family protein | Mobile elements | PE143B_0103415 | -6.9 |
|  | Mobile element protein | Mobile elements | PE143B_0105795 | Not present at 8°C |
|  | Phage tail length tape-measure protein | Mobile elements | PE143B_0109850 | -5.5 |
|  | Tn7-like transposition protein D | Mobile elements | PE143B_0114555 | -4.8 |
|  | Mobile element protein | Mobile elements | PE143B_0114885 | -3.1 |
|  | Transcriptional regulator. Cro/CI family | Mobile elements | PE143B_0115090 | -5.5 |
|  | Mobile element protein | Mobile elements | PE143B_0117675 | -5.1 |
|  | Mobile element protein | Mobile elements | PE143B_0117680 | -7.9 |
|  | ISPsy5. transposase | Mobile elements | PE143B_0117685 | -6.3 |
|  | Phage integrase | Mobile elements | PE143B_0124970 | -3.0 |
| *tnsB* | Transposon Tn7 transposition protein tnsB | Mobile elements | PE143B_0128485 | -5.5 |
|  | Phage protein U | Mobile elements | PE143B_0128845 | -9.0 |
|  | Baseplate assembly protein V | Mobile elements | PE143B_0128855 | -22.0 |
|  | Pyocin R2_PP. holin | Mobile elements | PE143B_0128865 | -5.8 |
|  | Transcriptional regulator. Cro/CI family | Mobile elements | PE143B_0128870 | -2.8 |
|  | Phage tail fiber protein | Mobile elements | No anotada en ncbi | -5.9 |
|  | Phage-related protein | Mobile elements | PE143B_0130535 | -66.1 |
|  | Putative bacteriophage protein | Mobile elements | PE143B_0130755 | -108.6 |
|  | Mobile element protein | Mobile elements | PE143B_0130815 | -3.2 |
|  | ISPsy22. transposase truncated | Mobile elements | PE143B_0130885 | -5.0 |
|  | Mobile element protein | Mobile elements | PE143B_0130915 | -5.5 |
|  | Mobile element protein | Mobile elements | PE143B_0130925 | -2.7 |
|  | Mobile element protein | Mobile elements | PE143B_0129285 | -15.2 |
|  |  |  |  |  |
| *flgG* | Flagellar basal-body rod protein FlgG | Motility | PE143B_0108600 | -2.8 |
| *flgJH* | Flagellar L-ring protein FlgH | Motility | PE143B_0108605 | -3.5 |
| *flgK* | Flagellar hook-associated protein FlgK | Motility | PE143B_0108620 | -2.7 |
| *flgL* | Flagellar hook-associated protein FlgL | Motility | PE143B_0108625 | -2.5 |
| *fleQ* | Flagellar regulatory protein FleQ | Motility | PE143B_0108660 | -2.7 |
| *fliF* | Flagellar M-ring protein FliF | Motility | PE143B_0108680 | -2.7 |
| *fliG* | Flagellar motor switch protein FliG | Motility | PE143B_0108685 | -2.5 |
| *fliM* | Flagellar motor switch protein FliM | Motility | PE143B_0108730 | -2.9 |
| *flhA* | Flagellar biosynthesis protein FlhA | Motility | PE143B_0108775 | -3.0 |
| *fleN* | Flagellar synthesis regulator FleN | Motility | PE143B_0108785 | -2.8 |
| *pilG* | twitching motility protein PilG | Motility | PE143B_0110755 | -2.9 |
| *fliL* | Flagellar biosynthesis protein FliL | Motility | PE143B_0111380 | -2.7 |
|  | MotA/TolQ/ExbB proton channel family protein | Motility | PE143B_0120045 | -2.5 |
| *flgE* | Flagellar hook protein FlgE | Motility | PE143B_0122280 | -4.2 |
|  | MotA/TolQ/ExbB proton channel family protein | Motility | PE143B_0124480 | -5.5 |
| *fimV* | Probable type IV pilus assembly FimV | Motility | PE143B_0124640 | -2.8 |
|  |  |  |  |  |
| *glyA* | Serine hydroxymethyltransferase | Nitrogen metabolism | PE143B_0101125 | -3.1 |
|  | Asparagine synthase. glutamine-hydrolyzing | Nitrogen metabolism | PE143B_0101325 | -5.8 |
| *asnB* | Asparagine synthetase. glutamine-hydrolyzing | Nitrogen metabolism | PE143B_0101400 | -10.1 |
| *dapB* | Dihydrodipicolinate reductase | Nitrogen metabolism | PE143B_0101740 | -2.7 |
| *carB* | Carbamoyl-phosphate synthase large chain | Nitrogen metabolism | PE143B_0101750 | -2.8 |
| *ilvB* | Acetolactate synthase large subunit | Nitrogen metabolism | PE143B_0101980 | -2.9 |
| *ilvN* | Acetolactate synthase small subunit | Nitrogen metabolism | PE143B_0101485 | -2.7 |
| *ilvC* | Ketol-acid reductoisomerase | Nitrogen metabolism | PE143B_0101990 | -2.2 |
| *hppD* | 4-hydroxyphenylpyruvate dioxygenase | Nitrogen metabolism | PE143B_0102520 | -5.8 |
|  | Butyryl-CoA dehydrogenase | Nitrogen metabolism | PE143B_0102680 | -5.2 |
| *hisB* | Imidazoleglycerol-phosphate dehydratase | Nitrogen metabolism | PE143B_0104155 | -2.1 |
| *hisA* | Phosphoribosylformimino-5-aminoimidazole carboxamide ribotide isomerase | Nitrogen metabolism | PE143B_0104170 | -3.0 |
| *hisF* | Imidazole glycerol phosphate synthase cyclase subunit | Nitrogen metabolism | PE143B_0104175 | -3.7 |
| *glnA* | Glutamine synthetase type I | Nitrogen metabolism | PE143B_0104245 | -2.4 |
| *hutH* | Putative histidine ammonia-lyase protein | Nitrogen metabolism | PE143B_0104705 | -6.9 |
| *glnE* | Glutamate-ammonia-ligase adenylyltransferase | Nitrogen metabolism | PE143B_0104850 | -4.1 |
| *ureG* | Urease accessory protein UreG | Nitrogen metabolism | PE143B_0105380 | -17.7 |
|  | Urease gamma subunit | Nitrogen metabolism | PE143B_0105470 | -11.8 |
| *dapD* | 2.3.4.5-tetrahydropyridine-2.6-dicarboxylate N-succinyltransferase | Nitrogen metabolism | PE143B_0106200 | -4.2 |
| *glnD* | Uridylyltransferase | Nitrogen metabolism | PE143B_0106230 | -3.9 |
| *map* | Methionine aminopeptidase | Nitrogen metabolism | PE143B_0106235 | -2.9 |
|  | Cysteine synthase | Nitrogen metabolism | PE143B_0106520 | -5.6 |
| *dapA* | Dihydrodipicolinate synthase | Nitrogen metabolism | PE143B_0106820 | -6.3 |
| *glnA* | Glutamine synthetase type I | Nitrogen metabolism | PE143B_0106945 | Not present at 8°C |
|  | Glutamine amidotransferase class-I | Nitrogen metabolism | PE143B_0106970 | -40.1 |
| *dapA* | Dihydrodipicolinate synthase | Nitrogen metabolism | PE143B_0107170 | -2.7 |
| *phhB* | Pterin-4-alpha-carbinolamine dehydratase | Nitrogen metabolism | PE143B_0108580 | -4.9 |
| *phhA* | Phenylalanine-4-hydroxylase | Nitrogen metabolism | PE143B_0108590 | -5.3 |
| *hmgA* | Homogentisate 1.2-dioxygenase | Nitrogen metabolism | PE143B_0109015 | -31.5 |
|  | Glutamate Aspartate periplasmic binding protein precursor GltI | Nitrogen metabolism | PE143B_0109455 | -3.9 |
| *serA* | D-3-phosphoglycerate dehydrogenase | Nitrogen metabolism | PE143B_0111110 | -2.6 |
|  | Glutamine synthetase family protein | Nitrogen metabolism | PE143B_0111255 | -3.0 |
|  | Aminomethyltransferase (glycine cleavage system T protein) | Nitrogen metabolism | PE143B_0111300 | -2.5 |
| *lyS* | Carboxynorspermidine dehydrogenase | Nitrogen metabolism | PE143B_0111640 | -4.0 |
| *metH* | 5-methyltetrahydrofolate--homocysteine methyltransferase | Nitrogen metabolism | PE143B_0112730 | -2.5 |
|  | Lysine decarboxylase 2 | Nitrogen metabolism | PE143B_0112815 | -3.7 |
|  | Branched-chain amino acid aminotransferase | Nitrogen metabolism | PE143B_0113205 | -2.7 |
| *mvaB* | Hydroxymethylglutaryl-CoA lyase | Nitrogen metabolism | PE143B_0113780 | -6.1 |
|  | Aspartate aminotransferase | Nitrogen metabolism | PE143B_0114335 | -3.3 |
|  | Creatinase | Nitrogen metabolism | PE143B_0114830 | -9.4 |
| *aspC* | Aspartate aminotransferase | Nitrogen metabolism | PE143B_0115335 | -4.1 |
| *leuA* | 2-isopropylmalate synthase | Nitrogen metabolism | PE143B_0115345 | -3.9 |
| *gabT* | 5-aminovalerate aminotransferase / Gamma-aminobutyrate:alpha-ketoglutarate aminotransferase | Nitrogen metabolism | PE143B_0116055 | -5.3 |
|  | Aminotransferase | Nitrogen metabolism | PE143B_0116465 | -11.0 |
|  | NAD-specific glutamate dehydrogenase | Nitrogen metabolism | PE143B_0116700 | -2.9 |
| *murI* | Glutamate racemase | Nitrogen metabolism | PE143B_0117295 | -7.9 |
| *trpB* | Tryptophan synthase beta chain | Nitrogen metabolism | PE143B_0118345 | -13.0 |
| *gmhB* | Histidinol-phosphatase | Nitrogen metabolism | PE143B_0118525 | -2.4 |
| *ahcY* | Adenosylhomocysteinase | Nitrogen metabolism | PE143B_0118820 | -4.1 |
|  | 2-keto-3-deoxy-D-arabino-heptulosonate-7-phosphate synthase I alpha | Nitrogen metabolism | PE143B_0119365 | -31.5 |
| *mmsA* | Methylmalonate-semialdehyde dehydrogenase | Nitrogen metabolism | PE143B_0120340 | -11.0 |
|  | Periplasmic aromatic amino acid aminotransferase beta precursor | Nitrogen metabolism | PE143B_0120655 | -12.0 |
|  | 5-aminopentanamidase | Nitrogen metabolism | PE143B_0120960 | -8.1 |
|  | Lysine 2-monooxygenase | Nitrogen metabolism | PE143B_0120965 | -5.8 |
|  | Aspartyl aminopeptidase | Nitrogen metabolism | PE143B_0121340 | -3.4 |
|  | 2-oxo-4-hydroxy-4-carboxy--5-ureidoimidazoline (OHCU) decarboxylase | Nitrogen metabolism | PE143B_0121395 | -9.2 |
| *serC* | Phosphoserine aminotransferase | Nitrogen metabolism | PE143B_0121880 | -2.8 |
| *ltaA/ltaE* | Low-specificity L-threonine aldolase | Nitrogen metabolism | PE143B_0122415 | -5.5 |
| *thrB* | Homoserine kinase | Nitrogen metabolism | PE143B_0122990 | -5.5 |
| *aspA* | Aspartate ammonia-lyase | Nitrogen metabolism | PE143B_0124315 | -6.0 |
| *leuC* | 3-isopropylmalate dehydratase large subunit | Nitrogen metabolism | PE143B_0124610 | -8.7 |
| *leuD* | 3-isopropylmalate dehydratase small subunit | Nitrogen metabolism | PE143B_0124615 | -53.5 |
| *ansA* | L-asparaginase | Nitrogen metabolism | PE143B_0124820 | -5.9 |
|  | Phosphoserine phosphatase | Nitrogen metabolism | PE143B_0124970 | -3.9 |
| *yfdZ* | PLP-dependent aminotransferase YfdZ | Nitrogen metabolism | PE143B_0125105 | -3.2 |
| *gshA* | Glutamate--cysteine ligase | Nitrogen metabolism | PE143B_0125350 | -5.6 |
|  | Aminotransferase. class III | Nitrogen metabolism | PE143B_0125520 | -3.4 |
| *dadA* | D-amino acid dehydrogenase small subunit | Nitrogen metabolism | PE143B_0125675 | -5.2 |
| *alr* | Alanine racemase | Nitrogen metabolism | PE143B_0125685 | -10.0 |
| *mmsA* | Methylmalonate-semialdehyde dehydrogenase | Nitrogen metabolism | PE143B_0128055 | -6.0 |
|  | Omega-amino acid--pyruvate aminotransferase | Nitrogen metabolism | PE143B_0128060 | -9.6 |
|  | 4-carboxymuconolactone decarboxylase | Nitrogen metabolism | PE143B_0128260 | -7.2 |
| *argC* | N-acetyl-gamma-glutamyl-phosphate reductase | Nitrogen metabolism | PE143B_0130130 | -2.9 |
|  | Biosynthetic Aromatic amino acid aminotransferase alpha | Nitrogen metabolism | PE143B_0130225 | -3.5 |
|  |  |  |  |  |
|  | Helicase related protein | Nucleic acid modification | PE143B_0100105 | -2.4 |
|  | Xanthine and CO dehydrogenases maturation factor | Nucleic acid modification | PE143B_0100210 | -3.5 |
|  | Ser-tRNA(Ala) deacylase; Gly-tRNA(Ala) deacylase | Nucleic acid modification | PE143B_0101140 | Not present at 8°C |
| *hsdR* | Type I restriction-modification system. restriction subunit R | Nucleic acid modification | PE143B_0101495 | -3.0 |
| *recC* | Exodeoxyribonuclease V gamma chain | Nucleic acid modification | PE143B_0101530 | -2.8 |
| *recB* | Exodeoxyribonuclease V beta chain | Nucleic acid modification | PE143B_0101530 | -3.8 |
| *recD* | Exodeoxyribonuclease V alpha chain | Nucleic acid modification | PE143B_0101535 | -4.3 |
|  | 5-methylcytosine-specific restriction enzyme McrB | Nucleic acid modification | PE143B_0101570 | -2.8 |
| *recN* | DNA repair protein RecN | Nucleic acid modification | PE143B_0101720 | -3.8 |
| *dinB* | dinB protein | Nucleic acid modification | PE143B_0102345 | -51.9 |
| *tnpT* | Cointegrate resolution protein T | Nucleic acid modification | PE143B_0102480 | -3.5 |
|  | tRNA (cytosine34-2'-O-)-methyltransferase | Nucleic acid modification | PE143B_0104230 | -5.7 |
| *argS* | Arginyl-tRNA synthetase | Nucleic acid modification | PE143B_0104540 | -5.3 |
| *parE* | Topoisomerase IV subunit B | Nucleic acid modification | PE143B_0105060 | -2.8 |
| *queG* | Epoxyqueuosine (oQ) reductase QueG | Nucleic acid modification | PE143B_0105130 | -4.7 |
| *mutL* | DNA mismatch repair protein MutL | Nucleic acid modification | PE143B_0105145 | -2.6 |
| *hsdR* | Type I restriction-modification system. restriction subunit R | Nucleic acid modification | PE143B_0105820 | -2.4 |
| *recA* | RecA protein | Nucleic acid modification | PE143B_0105860 | -2.7 |
| *lysS* | Lysyl-tRNA synthetase (class II) | Nucleic acid modification | PE143B_0106025 | -2.5 |
| *rapA* | RNA polymerase associated protein RapA | Nucleic acid modification | PE143B_0106610 | -3.5 |
|  | RNA methyltransferase. TrmH family | Nucleic acid modification | PE143B_0107340 | -6.5 |
| *alaS* | Alanyl-tRNA synthetase domain protein | Nucleic acid modification | PE143B_0107830 | -34.6 |
| *rraA* | Ribonuclease E inhibitor RraA | Nucleic acid modification | PE143B_0107875 | -3.6 |
|  | Methionyl-tRNA synthetase | Nucleic acid modification | PE143B_0109580 | -4.2 |
|  | Adenosine (5')-pentaphospho-(5'')-adenosine pyrophosphohydrolase | Nucleic acid modification | PE143B_0111070 | -2.4 |
| *dnaQ* | DNA polymerase III epsilon subunit | Nucleic acid modification | PE143B_0112820 | -3.5 |
| *queA* | S-adenosylmethionine:tRNA ribosyltransferase-isomerase | Nucleic acid modification | PE143B_0115205 | -2.6 |
|  | tRNA:Cm32/Um32 methyltransferase | Nucleic acid modification | PE143B_0115240 | -4.4 |
|  | SSU ribosomal protein S16p | Nucleic acid modification | PE143B_0115495 | -2.1 |
|  | Resolvase | Nucleic acid modification | PE143B_0116030 | -6.2 |
|  | Peptide chain release factor 3 | Nucleic acid modification | PE143B_0116880 | -2.4 |
| *ileS* | Isoleucyl-tRNA synthetase | Nucleic acid modification | PE143B_0117160 | -3.2 |
|  | Glutamyl-tRNA reductase | Nucleic acid modification | PE143B_0117315 | -3.6 |
| *rsmH* | rRNA small subunit methyltransferase H | Nucleic acid modification | PE143B_0117630 | -2.7 |
| *def* | Peptide deformylase | Nucleic acid modification | PE143B_0118440 | -3.2 |
| *glyQ* | Glycyl-tRNA synthetase alpha chain | Nucleic acid modification | PE143B_0118475 | -4.7 |
| *glyS* | Glycyl-tRNA synthetase beta chain | Nucleic acid modification | PE143B_0118480 | -2.3 |
| *dnaA* | Chromosomal replication initiator protein | Nucleic acid modification | PE143B_0118535 | -2.5 |
| *uvrD* | ATP-dependent DNA helicase UvrD/PcrA | Nucleic acid modification | PE143B_0118775 | -4.0 |
|  |  |  |  |  |
| *aspS* | Aspartyl-tRNA synthetase | Nucleic acid modification | PE143B_0120015 | -3.0 |
| *wbpM* | Nucleoside-diphosphate sugar epimerase/dehydratase | Nucleic acid modification | PE143B_0121995 | -3.2 |
| *rne* | Ribonuclease E | Nucleic acid modification | PE143B_0122220 | -2.8 |
|  | Ribonucleotide reductase of class Ia (aerobic). alpha subunit | Nucleic acid modification | PE143B_0122275 | -2.4 |
| *alaS* | Alanyl-tRNA synthetase | Nucleic acid modification | PE143B_0122410 | -2.8 |
|  | Holliday junction resolvasome. helicase subunit | Nucleic acid modification | PE143B_0123130 | -16.9 |
| *rplQ* | LSU ribosomal protein L17p | Nucleic acid modification | PE143B_0123710 | -2.1 |
| *truA* | tRNA pseudouridine synthase A | Nucleic acid modification | PE143B_0124650 | -3.7 |
| *purF* | Amidophosphoribosyltransferase | Nucleic acid modification | PE143B_0124680 | -3.6 |
| *pheS* | Phenylalanyl-tRNA synthetase alpha chain | Nucleic acid modification | PE143B_0124930 | -2.9 |
|  | LSU ribosomal protein L28p | Nucleic acid modification | PE143B_0125620 | -2.4 |
|  | LSU ribosomal protein L33p | Nucleic acid modification | PE143B_0125625 | -2.2 |
|  | endoribonuclease L-PSP family protein | Nucleic acid modification | PE143B_0125680 | -7.1 |
| *rep* | ATP-dependent DNA helicase Rep | Nucleic acid modification | PE143B_0125710 | -3.4 |
|  | DNA recombination-dependent growth factor C | Nucleic acid modification | PE143B_0127505 | -2.9 |
| *gatB* | Aspartyl-tRNA amidotransferase subunit B | Nucleic acid modification | PE143B_0128275 | -3.6 |
| *gatA* | Aspartyl-tRNA amidotransferase subunit A | Nucleic acid modification | PE143B_0128280 | -3.8 |
| *gatC* | Aspartyl-tRNA amidotransferase subunit C | Nucleic acid modification | PE143B_0128285 | -3.3 |
|  | tRNA-2-thiocytidine synthetase | Nucleic acid modification | PE143B_0129665 | -5.9 |
| *gltX* | Glutamyl-tRNA synthetase | Nucleic acid modification |  | -2.9 |
|  | DNA exonuclease X | Nucleic acid modification | PE143B_0130750 | -54.7 |
|  |  |  |  |  |
|  | Catechol 2.3-dioxygenase | Oxygenase | PE143B_0103495 | -44.1 |
| *lpxO* | Fe(2+)/alpha-ketoglutarate-dependent dioxygenase LpxO | Oxygenase | PE143B_0107420 | -5.4 |
| *alkB* | Alkane-1 monooxygenase | Oxygenase | PE143B_0112385 | -7.5 |
|  | 4-hydroxybenzoyl-CoA thioesterase family active site | Oxygenase | PE143B_0120040 | -2.2 |
| *ntaB* | Nitrilotriacetate monooxygenase component B | Oxygenase | PE143B_0122655 | -2.5 |
|  | Flavin-containing monooxygenase | Oxygenase | PE143B_0122745 | -2.1 |
|  | Dioxygenases related to 2-nitropropane dioxygenase | Oxygenase | PE143B_0130120 | -8.3 |
|  |  |  |  |  |
| *hslU* | ATP-dependent hsl protease ATP-binding subunit HslU | Protease | PE143B_0104525 | -7.1 |
| *hslV* | ATP-dependent protease HslV | Protease | PE143B_0104530 | -4.7 |
|  | Membrane-associated zinc metalloprotease | Protease | PE143B_0106275 | -2.5 |
| *lon* | ATP-dependent protease La. Type I | Protease | PE143B_0109210 | -9.9 |
| *tatD* | Deoxyribonuclease TatD | Protease | PE143B_0114535 | -13.2 |
|  | Lipoprotein signal peptidase | Protease | PE143B_0117155 | -2.6 |
|  | Periplasmic serine proteases (ClpP class) | Protease | PE143B_0122205 | -2.4 |
|  | Oligopeptidase A | Protease | PE143B_0122860 | -5.5 |
| *tldD* | TldD protein. part of proposed TldE/TldD proteolytic complex | Protease | PE143B_0128325 | -2.7 |
| *pepN* | Aminopeptidase N | Protease | PE143B_0128475 | -2.8 |
| *clpA* | ATP-dependent Clp protease ATP-binding subunit ClpA | Protease | PE143B_0129570 | -2.4 |
|  | Peptidase M16 | Protease | PE143B_0110900 | -6.2 |
|  | Transcriptional regulator. TetR family | Regulator protein | PE143B_0100275 | -17.1 |
| *putR* | Regulator PutR for proline utilization | Regulator protein | PE143B_0101185 | -3.5 |
| *greA* | Transcription elongation factor GreA | Regulator protein | PE143B_0101755 | -2.3 |
|  | C4-type zinc finger protein. DksA/TraR | Regulator protein | PE143B_0101935 | -3.2 |
| *cyaA* | Adenylate cyclase | Regulator protein | PE143B_0102055 | -16.5 |
|  | Diguanylate cyclase | Regulator protein | PE143B_0102180 | -4.9 |
|  | MvaT-like transcriptional regulator | Regulator protein | PE143B_0102510 | -3.0 |
|  | Transcriptional regulators | Regulator protein | PE143B_0103525 | -6.8 |
|  | Helix-turn-helix motif | Regulator protein | No anotada en ncbi | -12.2 |
| *putA* | Transcriptional repressor of PutA and PutP | Regulator protein | PE143B_0104800 | -2.2 |
|  | Sensory box/GGDEF family protein | Regulator protein | PE143B_0104835 | -2.9 |
|  | Predicted signal transduction protein | Regulator protein | PE143B_0105100 | -3.4 |
| *csiR* | CsiR. transcriptional repressor of CsiD | Regulator protein | PE143B_0106900 | -3.1 |
| *csiD* | Carbon starvation induced protein CsiD | Regulator protein | PE143B_0106905 | -18.9 |
| *tctD* | Tricarboxylate transport transcriptional regulator TctD | Regulator protein | PE143B_0107090 | -10.5 |
| *ybhD* | LysR family transcriptional regulator YbhD | Regulator protein | PE143B_0107200 | -31.5 |
|  | Transcriptional regulator. LysR family | Regulator protein | PE143B_0107240 | -9.8 |
|  | Methyl-accepting chemotaxis protein I (serine chemoreceptor protein) | Regulator protein | PE143B_0107680 | -2.5 |
| *gbuR* | Transcriptional regulator GbuR | Regulator protein | PE143B_0108340 | -6.9 |
|  | Transcriptional regulator. IclR family | Regulator protein | PE143B_0109010 | Not present at 8°C |
|  | Component of chemotactic signal transduction system | Regulator protein | PE143B_0110735 | -2.9 |
| *rpoH* | RNA polymerase sigma factor RpoH | Regulator protein | PE143B_0110880 | -2.8 |
|  | Transcriptional regulator. GntR family | Regulator protein | PE143B_0111420 | -12.6 |
|  | DNA-binding response regulator | Regulator protein | PE143B_0113050 | -9.1 |
|  | sensor histidine kinase | Regulator protein | PE143B_0113055 | -6.5 |
|  | Methyl-accepting chemotaxis protein I (serine chemoreceptor protein) | Regulator protein | PE143B_0114540 | -8.5 |
| *IscR* | Iron-sulfur cluster regulator IscR | Regulator protein | PE143B_0115250 | -2.2 |
|  | Sigma factor. ECF subfamily | Regulator protein | PE143B_0115735 | -5.1 |
|  | Transcriptional regulator. IclR family | Regulator protein | PE143B_0115965 | -5.0 |
| *zraR* | Transcriptional regulatory protein zraR | Regulator protein | PE143B_0118105 | -31.5 |
|  | Helix-turn-helix. AraC type | Regulator protein | PE143B_0118165 | -12.4 |
| *gmlR* | Transcriptional regulator of glmS gene | Regulator protein | PE143B_0118615 | -5.8 |
| *hexR* | Phosphogluconate repressor HexR | Regulator protein | PE143B_0118785 | -5.9 |
|  | Transcriptional regulator. MerR family | Regulator protein | PE143B_0119430 | -12.6 |
|  | Transcriptional regulator. LysR family | Regulator protein | PE143B_0119480 | -4.9 |
|  | Transmembrane sensor | Regulator protein | PE143B_0120800 | -4.9 |
|  | RNA polymerase sigma-70 factor. ECF subfamily | Regulator protein | PE143B_0120805 | -8.3 |
|  | Transcriptional regulator. GntR family | Regulator protein | PE143B_0121425 | -8.9 |
|  | Methyl-accepting chemotaxis protein | Regulator protein | PE143B_0122650 | -2.3 |
|  | Transcriptional regulator. MerR family | Regulator protein | PE143B_0122695 | -2.4 |
| *dnr* | Nitric oxide -responding transcriptional regulator Dnr (Crp/Fnr family) | Regulator protein |  | -2.4 |
|  | Predicted signal transduction protein | Regulator protein | PE143B_0124455 | -3.8 |
|  | Transcription accessory protein (S1 RNA-binding domain) | Regulator protein | PE143B_0125360 | -3.0 |
| *cyaA* | Adenylate cyclase | Regulator protein | PE143B_0125820 | -4.5 |
| *cheB* | Chemotaxis response regulator protein-glutamate methylesterase CheB | Regulator protein | PE143B_0126040 | -15.7 |
|  | Methyl-accepting chemotaxis protein I (serine chemoreceptor protein) | Regulator protein | PE143B_0126060 | -3.7 |
|  | Transcriptional regulator of the arabinose operon in Shewanella. GntR family | Regulator protein | PE143B_0126490 | -9.4 |
|  | Methyl-accepting chemotaxis protein | Regulator protein | PE143B_0126985 | -2.3 |
|  | Transcriptional regulator. TetR family | Regulator protein | PE143B_0127275 | -10.2 |
|  | Sensory box/GGDEF family protein | Regulator protein | PE143B_0127345 | -6.0 |
|  | Transcriptional regulator. MarR family | Regulator protein | PE143B_0127735 | -22.0 |
|  | Transcriptional regulator. TetR family | Regulator protein | PE143B_0127805 | -7.2 |
|  | LacI family transcriptional regulator | Regulator protein | PE143B_0127975 | -7.3 |
| *pfeR* | Two-component response regulator PfeR. enterobactin | Regulator protein | PE143B_0128935 | -16.5 |
| *pfeS* | Two-component sensor histidine kinase PfeS. enterobactin | Regulator protein | PE143B_0128940 | -6.8 |
|  | Transmembrane sensor | Regulator protein | PE143B_0129885 | -7.0 |
|  |  |  |  |  |
| *arnT* | 4-amino-4-deoxy-L-arabinose transferase | Resistance to antibiotics and metals | PE143B_0101285 | -6.3 |
|  | Fusaric acid resistance protein | Resistance to antibiotics and metals | PE143B_0100295 | -4.6 |
|  | Cobalt-zinc-cadmium resistance protein | Resistance to antibiotics and metals | PE143B_0102175 | -58.2 |
|  | MbtH-like protein | Resistance to antibiotics and metals | PE143B_0121325 | -18.1 |
| *terC* | Integral membrane protein TerC | Resistance to antibiotics and metals | PE143B_0123110 | -4.5 |
|  | Colicin V production protein | Resistance to antibiotics and metals | PE143B_0124675 | -2.6 |
|  | Isochorismatase | Resistance to antibiotics and metals | PE143B_0127025 | -4.5 |
|  | Acriflavin resistance plasma membrane protein | Resistance to antibiotics and metals | PE143B_0127360 | -3.1 |
|  |  |  |  |  |
| *ahpC* | Alkyl hydroperoxide reductase protein C | Stress resistance | PE143B_0102490 | -11.1 |
| *ahpF* | Alkyl hydroperoxide reductase protein F | Stress resistance | PE143B_0102495 | -18.9 |
| *kefA* | Potassium efflux system KefA protein / Small-conductance mechanosensitive channel | Stress resistance | PE143B_0104770 | -3.1 |
|  | Na(+)/H(+) antiporter | Stress resistance | PE143B_0104775 | -4.1 |
| *psiF* | Phosphate starvation-inducible protein psiF precursor | Stress resistance | PE143B_0105425 | -6.8 |
|  | S-formylglutathione hydrolase | Stress resistance | PE143B_0106365 | -3.6 |
|  | Universal stress protein family | Stress resistance | PE143B_0107470 | -3.7 |
| *cmpX* | CmpX | Stress resistance | PE143B_0107890 | -2.4 |
|  | Glutaredoxin-related protein | Stress resistance | PE143B_0109500 | -2.2 |
|  | Alkyl hydroperoxide reductase subunit C-like protein | Stress resistance | PE143B_0109515 | -3.3 |
| *nfuA* | NfuA Fe-S protein maturation | Stress resistance | PE143B_0112720 | -2.1 |
|  | Glutathione peroxidase | Stress resistance | PE143B_0114325 | -7.2 |
| *suhB* | Inositol-1-monophosphatase | Stress resistance | PE143B_0115235 | -2.7 |
| *rosB* | Potassium/proton antiporter ROSB | Stress resistance | PE143B_0118760 | -5.8 |
| *glpE* | Thiosulfate sulfurtransferase GlpE | Stress resistance | PE143B_0121060 | -9.9 |
|  | Organic solvent tolerance protein precursor | Stress resistance | PE143B_0121090 | -2.2 |
|  | Glutathione S-transferase | Stress resistance | PE143B_0121430 | -4.8 |
| *soxG1* | Sarcosine oxidase gamma subunit | Stress resistance | PE143B_0122665 | -2.5 |
| *soxA1* | Sarcosine oxidase alpha subunit | Stress resistance | PE143B_0122670 | -2.5 |
| *soxD1* | Sarcosine oxidase delta subunit | Stress resistance | PE143B_0122675 | -2.4 |
| *soxB1* | Sarcosine oxidase beta subunit | Stress resistance | PE143B_0122680 | -2.4 |
| *soxA2* | Sarcosine oxidase alpha subunit | Stress resistance |  | -2.0 |
| *soxA3* | Sarcosine oxidase alpha subunit | Stress resistance | PE143B_0122755 | -2.1 |
|  | Carbonic anhydrase. family 3 | Stress resistance | PE143B_0122855 | -4.8 |
| *fam* | Trans-aconitate 2-methyltransferase | Stress resistance | PE143B_0123680 | -13.8 |
| *oxyR* | Hydrogen peroxide-inducible genes activator. OxyR | Stress resistance | PE143B_0124465 | -4.1 |
| *SpoT* | GTP pyrophosphokinase. (p)ppGpp synthetase II | Stress resistance | PE143B_0124500 | -2.3 |
| *sod* | Superoxide dismutase [Fe] | Stress resistance | PE143B_0125285 | -2.3 |
| *dsbB* | Periplasmic thiol:disulfide oxidoreductase DsbB. required for DsbA reoxidation | Stress resistance | PE143B_0125870 | -13.0 |
|  | Rhodanese | Stress resistance | PE143B_0127170 | -2.5 |
|  | Calcium/proton antiporter | Stress resistance | PE143B_0128095 | -9.0 |
|  |  |  |  |  |
| *aceF* | Dihydrolipoamide acetyltransferase component of pyruvate dehydrogenase complex | TCA | PE143B_0104840 | -3.5 |
| *aceE* | Pyruvate dehydrogenase E1 component | TCA | PE143B_0104845 | -4.0 |
| *lhgO* | L-2-hydroxyglutarate oxidase | TCA | PE143B_0106910 | -4.3 |
| *ppsA* | Phosphoenolpyruvate synthase | TCA | PE143B_0107865 | -2.5 |
|  | Succinyl-CoA ligase [ADP-forming] alpha chain | TCA | PE143B_0114010 | -2.4 |
|  | Dihydrolipoamide dehydrogenase of 2-oxoglutarate dehydrogenase | TCA | PE143B_0114020 | -2.3 |
| *sucB* | Dihydrolipoamide succinyltransferase component (E2) of 2-oxoglutarate dehydrogenase complex | TCA | PE143B_0114025 | -2.7 |
| *sucA* | 2-oxoglutarate dehydrogenase E1 component | TCA | PE143B_0114030 | -2.0 |
| *sdhB* | Succinate dehydrogenase iron-sulfur protein | TCA | PE143B_0114035 | -2.2 |
| *sdhD* | Succinate dehydrogenase hydrophobic membrane anchor protein | TCA | PE143B_0114045 | -2.7 |
|  | Succinate dehydrogenase cytochrome b-556 subunit | TCA | PE143B_0114050 | -2.1 |
|  | Aconitate hydratase/ 2-methylisocitrate dehydratase | TCA | PE143B_0116660 | -3.4 |
|  | Pyruvate carboxyl transferase subunit A | TCA | PE143B_0118795 | -11.0 |
|  | Pyruvate carboxyl transferase subunit B | TCA | PE143B_0118800 | -6.6 |
| *idh* | Isocitrate dehydrogenase [NADP]/ Monomeric isocitrate dehydrogenase | TCA | PE143B_0123945 | -2.6 |
| *aceA* | Isocitrate lyase | TCA | PE143B_0123985 | -2.9 |
|  |  |  |  |  |
|  | Multidrug resistance protein B | Transport family protein | PE143B_0100165 | -3.6 |
|  | Leucine-responsive regulatory protein. regulator for leucine (or lrp) regulon and high-affinity branched-chain amino acid transport system | Transport family protein | PE143B_0100950 | -8.1 |
|  | ABC transporter. ATP-binding protein | Transport family protein | PE143B_0101195 | -3.5 |
| *lldP* | L-lactate permease | Transport family protein | PE143B_0101680 | -19.4 |
| *modA* | Molybdenum ABC transporter. periplasmic molybdenum-binding protein ModA | Transport family protein | PE143B_0102420 | -8.4 |
| *potG* | Putrescine transport ATP-binding protein PotG | Transport family protein | PE143B_0103840 | -30.7 |
| *potI* | Putrescine transport system permease protein PotI | Transport family protein | PE143B_0103850 | -6.6 |
|  | Amino acid ABC transporter. periplasmic amino acid-binding protein | Transport family protein | PE143B_0103965 | -3.5 |
|  | Transport permease protein of gamma-aminobutyrate | Transport family protein | PE143B_0103975 | -7.5 |
| *braG* | Branched-chain amino acid ABC transporter | Transport family protein | PE143B_0105325 | -9.4 |
|  | ABC transporter | Transport family protein | PE143B_0105825 | -2.9 |
| *cmaX* | CmaX protein | Transport family protein | PE143B_0107880 | -3.2 |
| *crfX* | CrfX protein | Transport family protein | PE143B_0107885 | -6.0 |
|  | Permease of the drug/metabolite transporter (DMT) superfamily | Transport family protein | PE143B_0108160 | -59.8 |
|  | D-serine/D-alanine/glycine transporter | Transport family protein | PE143B_0109280 | -13.7 |
| *gltL* | Glutamate Aspartate transport ATP-binding protein GltL | Transport family protein | PE143B_0109440 | -3.1 |
| *gltK* | Glutamate Aspartate transport system permease protein GltK | Transport family protein | PE143B_0109445 | -4.3 |
| *glpF* | Glycerol uptake facilitator protein | Transport family protein | PE143B_0109475 | -5.3 |
| *potH* | Putrescine transport system permease protein PotH | Transport family protein | PE143B_0111165 | -2.9 |
| *potG* | Putrescine transport ATP-binding protein PotG | Transport family protein | PE143B_0111170 | -3.0 |
| *potF* | Putrescine ABC transporter putrescine-binding protein PotF | Transport family protein | PE143B_0111175 | -5.2 |
|  | ABC-type multidrug transport system. permease component | Transport family protein | PE143B_0111375 | -9.4 |
| *hisJ* | Histidine transporter. periplasmic histidine-binding protein | Transport family protein | PE143B_0112950 | -3.6 |
|  | Membrane fusion component of tripartite multidrug resistance system | Transport family protein | PE143B_0113935 | -8.5 |
| *oprD* | Outer membrane porin. OprD | Transport family protein | PE143B_0114565 | -5.8 |
| *codB* | Cytosine permease | Transport family protein | PE143B_0114890 | -13.6 |
|  | ABC-type amino acid transport. periplasmic component | Transport family protein | PE143B_0114995 | -11.1 |
|  | Cation ABC transporter. periplasmic protein | Transport family protein | PE143B_0115165 | -5.7 |
| *cysW* | Sulfate transport system permease protein CysW | Transport family protein | PE143B_0116095 | -7.9 |
|  | Cystine ABC transporter. ATP-binding protein | Transport family protein | PE143B_0116290 | -6.5 |
| *fliY* | Cystine ABC transporter. periplasmic cystine-binding protein FliY | Transport family protein | PE143B_0116300 | -2.9 |
|  | Membrane fusion component of tripartite multidrug resistance system | Transport family protein | PE143B_0117480 | Not present at 8°C |
|  | L-lysine permease | Transport family protein | PE143B_0117760 | -4.6 |
|  | ABC transporter protein. ATP binding component | Transport family protein | PE143B_0117925 | -15.0 |
|  | ABC-type amino acid transport. periplasmic component | Transport family protein | PE143B_0118935 | -13.6 |
|  | Permeases of the major facilitator superfamily | Transport family protein | PE143B_0118955 | -6.2 |
| *mgtA* | Mg(2+) transport ATPase. P-type | Transport family protein | PE143B_0119390 | -4.2 |
|  | RhtB family transporter | Transport family protein | PE143B_0119510 | -22.8 |
|  | Dicarboxylate MFS transporter | Transport family protein | PE143B_0119825 | -4.9 |
|  | Putrescine transport ATP-binding protein PotA | Transport family protein | PE143B_0121125 | -5.7 |
|  | ABC transporter. periplasmic spermidine putrescine-binding protein PotD | Transport family protein | PE143B_0121130 | -7.3 |
|  | Spermidine Putrescine ABC transporter permease component PotB | Transport family protein | PE143B_0121135 | -6.6 |
|  | Spermidine Putrescine ABC transporter permease component PotC | Transport family protein | PE143B_0121140 | -23.6 |
|  | Aerobic C4-dicarboxylate transporter for fumarate. L-malate. D-malate, succinate, aspartate | Transport family protein | PE143B_0122235 | -73.6 |
|  | Cytosine/purine/uracil/thiamine/allantoin permease family protein | Transport family protein | PE143B_0122660 | -2.2 |
|  | Amino acid permease | Transport family protein | PE143B_0122735 | -2.1 |
|  | Sulfate transporter family protein in cluster with carbonic anhydrase | Transport family protein | PE143B_0122885 | -7.9 |
|  | Outer membrane protein W precursor | Transport family protein | PE143B_0123645 | -117.0 |
|  | Biopolymer transport protein ExbD/TolR | Transport family protein | PE143B_0124475 | -2.8 |
|  | Outer membrane porin. OprD family | Transport family protein | PE143B_0125535 | -5.7 |
|  | RND transporter. membrane fusion protein | Transport family protein | PE143B_0127365 | -3.2 |
|  | D-galactonate transporter | Transport family protein | PE143B_0127980 | -25.7 |
|  | Protein co-occurring with transport systems | Transport family protein | PE143B_0128080 | -7.9 |
|  | Putative TEGT family carrier/transport protein | Transport family protein | PE143B_0128990 | -2.9 |
|  | ABC transporter. transmembrane region. bacteriocin processing | Transport family protein | PE143B_0129400 | -2.8 |
|  |  |  |  |  |
| *thiE* | Thiamin-phosphate pyrophosphorylase | Vitamins and cofactors | PE143B_0100715 | -5.0 |
| *accC* | Biotin carboxylase of acetyl-CoA carboxylase | Vitamins and cofactors | PE143B_0105625 | -2.3 |
| *bioA* | Adenosylmethionine-8-amino-7-oxononanoate aminotransferase | Vitamins and cofactors | PE143B_0110620 | -3.7 |
| *folD-2* | Methylenetetrahydrofolate dehydrogenase (NADP+) /cyclohydrolase | Vitamins and cofactors | PE143B_0113510 | -3.7 |
| *mobA* | Molybdopterin-guanine dinucleotide biosynthesis protein MobA | Vitamins and cofactors | PE143B_0114230 | -9.8 |
| *thiF* | Sulfur carrier protein adenylyltransferase ThiF | Vitamins and cofactors | PE143B_0114665 | -3.6 |
| *mobB* | Molybdopterin biosynthesis protein MobB | Vitamins and cofactors | PE143B_0117300 | -4.5 |
| *atpB* | ATP synthase A chain | Vitamins and cofactors | PE143B_0118570 | -2.1 |
| *atpE* | ATP synthase C chain | Vitamins and cofactors | PE143B_0118575 | -2.2 |
| *metF* | 5.10-methylenetetrahydrofolate reductase | Vitamins and cofactors | PE143B_0118815 | -3.0 |
| *folX* | Dihydroneopterin triphosphate epimerase | Vitamins and cofactors | PE143B_0120520 | -2.2 |
|  | Undecaprenyl-phosphate N-acetylglucosaminyl 1-phosphate transferase | Vitamins and cofactors | PE143B_0121990 | -3.7 |
| *folD1* | Methylenetetrahydrofolate dehydrogenase (NADP+) / cyclohydrolase | Vitamins and cofactors | PE143B_0122680 | -2.3 |
| *purU* | Formyltetrahydrofolate deformylase | Vitamins and cofactors |  | -2.2 |
|  | Formyltetrahydrofolate deformylase | Nucleic acid modification | PE143B_0119905 | -3.8 |
|  | Methylenetetrahydrofolate dehydrogenase (NADP+)/ cyclohydrolase | Vitamins and cofactors | PE143B_0122730 | -2.2 |
|  | Formyltetrahydrofolate deformylase | Vitamins and cofactors |  | -2.1 |
|  | 1-deoxy-D-xylulose 5-phosphate synthase | Vitamins and cofactors | PE143B_0123540 | -2.7 |
|  | Outer membrane vitamin B12 receptor BtuB | Vitamins and cofactors | PE143B_0123545 | -4.8 |
|  | Adenylosuccinate lyase | Vitamins and cofactors | PE143B_0123965 | -3.0 |
|  |  |  |  |  |
