# Supplementary material for: Novel Essential Role of Ethanol Oxidation Genes at Low Temperature Revealed by Transcriptome Analysis in the Antarctic Bacterium Pseudomonas extremaustralis
Source: PLoS One. 2015 Dec 15;10(12):e0145353. doi: 10.1371/journal.pone.0145353 (PMC4686015; doi:10.1371/journal.pone.0145353)
Supplement: S2 Table — All genes presented P≤0.05 and Q≤0.05. (Rockhopper software). (DOC) [file pone.0145353.s006.doc]

**S2 Table. Up-regulated genes under cold conditions in *P. extremaustralis*. All genes presented P≤0.05 and Q≤0.05. (Rockhopper software).**

| **Gene** | **Function** | **Category** | **Locus-tag** | **Fold change** |
| --- | --- | --- | --- | --- |
| *gbuA* | Agmatinase | Arginine metabolism | PE143B_0108335 | 2.2 |
|  |  |  |  |  |
| *ivy* | Inhibitor of vertebrate lysozyme precursor | Cell wall and membrane | PE143B_0123060 | 3.0 |
| *pcs* | Phosphatidylcholine synthase | Cell wall and membrane | PE143B_0130015 | 6.0 |
|  |  |  |  |  |
|  | Cell division inhibitor | Cellular division | PE143B_0102280 | 3.8 |
|  |  |  |  |  |
| *cspA* | Cold shock protein CspA | Chaperone | PE143B_0106405 | 8.0 |
|  |  |  |  |  |
|  | Oxidoreductase | Cytochrome, quinone and oxidoreductase proteins | PE143B_0100505 | 2.1 |
| *exaB* | Cytochrome c550. associated with quino(hemo)protein alcohol dehydrogenase | Cytochrome, quinone and oxidoreductase proteins  Ethanol metabolism | PE143B_0113310 | 10.9 |
|  |  |  |  |  |
| *exaA1* | Ethanol dehydrogenase. PQQ-dependent | Ethanol metabolism | PE143B_0113330 | 7.6 |
| *exaC* | Aldehyde dehydrogenase | Ethanol metabolism | PE143B_0113335 | 8.7 |
|  |  |  |  |  |
| *algD* | GDP-mannose 6-dehydrogenase | Exopolysaccharide | PE143B_0117405 | 3.6 |
|  | Hypothetical protein | HP | PE143B_0101605 | 4.4 |
|  | Hypothetical protein | HP | PE143B_0101765 | 2.7 |
|  | Hypothetical protein | HP | PE143B_0127065 | 9.8 |
|  | Hypothetical protein | HP | PE143B_0126150 | 2.8 |
|  | Hypothetical protein | HP | PE143B_0125445 | 2.3 |
|  | Hypothetical protein | HP | PE143B_0125235 | 27.4 |
|  | Hypothetical protein | HP | PE143B_0122835 | 2.7 |
|  | Hypothetical protein | HP | PE143B_0123050 | Not present at 30°C |
|  | Hypothetical protein | HP | PE143B_0123055 | 4.1 |
|  | Hypothetical protein | HP | PE143B_0121785 | 5.6 |
|  | Hypothetical protein | HP | PE143B_0116505 | 5.4 |
|  | Hypothetical protein | HP | PE143B_0116795 | 2.7 |
|  | Hypothetical protein | HP | PE143B_0117965 | 5.4 |
|  | Hypothetical protein | HP | PE143B_0114090 | 4.6 |
|  | Hypothetical protein | HP | PE143B_0115245 | 4.6 |
|  | Hypothetical protein | HP | PE143B_0111505 | 7.5 |
|  | Hypothetical protein | HP | PE143B_0111510 | 8.1 |
|  | Lipoprotein YcfM. part of a salvage pathway of unknown substrate | HP | PE143B_0111515 | 4.9 |
|  | Hypothetical protein | HP | PE143B_0111520 | 15.3 |
|  | Hypothetical protein | HP | PE143B_0112325 | 13.3 |
|  | Hypothetical protein | HP | PE143B_0112965 | 2.3 |
|  | Hypothetical protein | HP | PE143B_0112970 | 6.7 |
|  | Hypothetical protein | HP | PE143B_0113245 | 8.6 |
|  | Hypothetical protein | HP | PE143B_0107970 | 3.1 |
|  | Hypothetical protein | HP | PE143B_0108285 | 5.3 |
|  | Hypothetical protein | HP | PE143B_0102955 | 16.5 |
|  | Hypothetical protein | HP | PE143B_0104095 | 7.0 |
|  | Hypothetical protein | HP | PE143B_0105280 | 3.8 |
|  | Hypothetical protein | HP | PE143B_0105905 | 4.4 |
|  | Hypothetical protein | HP | PE143B_0102395 | 4.6 |
|  | Hypothetical protein | HP | PE143B_0102400 | 2.0 |
|  | Hypothetical protein | HP | PE143B_0102465 | 2.5 |
|  | Hypothetical protein | HP | PE143B_0102600 | 25.4 |
|  |  |  |  |  |
| *bfrB* | Bacterioferritin | Iron and pyoverdin | PE143B_0109505 | 2.1 |
|  |  |  |  |  |
| *metE* | Homocysteine S-methyltransferase | Nitrogen metabolism | PE143B_0124260 | 1.8 |
|  | ADP-ribosylglycohydrolase | Nitrogen metabolism | PE143B_0119790 | 4.1 |
|  |  |  |  |  |
|  | Response regulator | Regulator protein | PE143B_0102610 | 4.2 |
| *slyA* | Transcriptional regulator SlyA | Regulator protein | PE143B_0108545 | 2.5 |
| *cpxR* | Copper-sensing two-component system response regulator CpxR | Regulator protein | PE143B_0120200 | 3.2 |
| *cheY* | Response regulator receiver (CheY-like protein) | Regulator protein | PE143B_0120695 | 4.7 |
| *algZ* | Alginate biosynthesis transcriptional activator | Regulator protein | PE143B_0122375 | 1.8 |
| *rsmE* | Carbon storage regulator | Regulator protein | PE143B_0124800 | 3.9 |
| *rnk* | Regulator of nucleoside diphosphate kinase | Regulator protein | PE143B_0125815 | 1.9 |
| *cheC* | Chemotaxis protein CheC -- inhibitor of MCP methylation | Regulator protein | PE143B_0126920 | 2.0 |
|  | Methyl-accepting chemotaxis protein | Regulator protein | PE143B_0127250 | 2.5 |
|  | Transcriptional regulator. TetR family | Regulator protein | PE143B_0127545 | 2.8 |
|  | Sensory box/GGDEF family protein | Regulator protein | PE143B_0118780 | 2.2 |
| *erbR* | Transcriptional regulator. LuxR family  Ethanol metabolism | Regulator protein | PE143B_0113255 | 2.4 |
|  |  |  |  |  |
|  | Rhodanese-related sulfurtransferase | Stress resistance | PE143B_0113260 | 8.1 |
|  | Sulfatase family protein | Stress resistance | PE143B_0115660 | 4.6 |
|  | Osmotically inducible protein Y precursor | Stress resistance | PE143B_0101915 | 5.3 |
|  | Sodium-solute symporter | Stress resistance | PE143B_0108320 | 6.6 |
|  |  |  |  |  |
|  | Amino acid ABC transporter. permease protein | Transport family protein | PE143B_0124250 | 2.4 |
| *oprB* | Glucose-selective porin OprB | Transport family proteins | PE143B_0125285 | 3.0 |
|  | ABC transporter. ATP-binding/permease protein | Transport family protein | PE143B_0127180 | 2.1 |
|  | Transport protein | Transport family protein | PE143B_0116790 | 2.3 |
