# Supplementary material for: Novel Essential Role of Ethanol Oxidation Genes at Low Temperature Revealed by Transcriptome Analysis in the Antarctic Bacterium Pseudomonas extremaustralis
Source: PLoS One. 2015 Dec 15;10(12):e0145353. doi: 10.1371/journal.pone.0145353 (PMC4686015; doi:10.1371/journal.pone.0145353)
Supplement: S3 Table — (DOC) [file pone.0145353.s007.doc]

| **Category**  **Over-represented** | **GO** |
| --- | --- |
| Polysaccharide metabolic process | GO:0005976 GO:0044723 GO:0016051 GO:0033692 GO:0034637 GO:0000271 GO:0044262 GO:0044264 |
| Lipid biosynthetic process | GO:0008610 GO:0044255 |
| Cellular macromolecule biosynthetic process | GO:0034645 GO:0009059  GO:1901576 GO:0044249 |
| Carbohydrate derivative metabolic process | GO:1901135 GO:1901137 |
| Organic cyclic compound biosynthetic process | GO:1901362 GO:0016070 |
| RNA biosynthetic process | GO:0032774 |
| Signaling | GO:0023052 GO:0060089 GO:0044700 GO:0007165 GO:0051716 |
| Lipopolysaccharide biosynthetic process | GO:1903509 GO:0008653 GO:0009103 |
| Lipopolysaccharide biosynthetic process | GO:1903509 GO:0008653 GO:0009103 |
| Aromatic compound biosynthetic process | GO:0019438 GO:0018130 |
| Nucleic acid-templated transcription | GO:0097659 |
| Transcription, DNA-templated(GO:0006351) |  |
| Regulation of biological process | GO:0050789 |
| Cellular amino acid metabolic process | GO:0006520 |
| Organic acid metabolic process | GO:0019752 GO:0006082 GO:0043436 |
| Small molecule metabolic process | GO:0044281 |
| Single-organism metabolic process | GO:0044710 |
| Organonitrogen compound metabolic process | GO:1901564 |
| Catabolic process | GO:0009056 |
| Generation of precursor metabolites and energy | GO:0006091 |
| Catalytic activity | GO:0003824 |
| Primary metabolic process | GO:0044238 |
| Organic substance metabolic process | GO:0071704 |
| Cytosol | GO:0005829 GO:0005737 |
| Single-organism cellular process | GO:0044763 |
| Single-organism process | GO:0044699 |

**S3 Table. GO categories in differentially expressed genes**
